# MENTSH: A novel mitochondrial microprotein linked to a SNP associated with type 2 diabetes

**DOI:** 10.7150/thno.134637

**Published:** 2026-07-20

**Authors:** Kelvin Yen, Ricardo Ramirez, Brendan Miller, Hiroshi Kumagai, Jie Yao, Morgan Levine, Xiuqing Guo, Jihui Sha, Ana Silverstein, Roberto Vicinanza, Kent D. Taylor, Melanie Flores, Zeferino Reyna, Su-Jeong Kim, Noel Guerrero, Hemal H. Mehta, Junxiang Wan, Zhongzheng Niu, Carrie V. Breton, Maria C. Kenney, Thalida Em Arpawong, James Wohlschlegel, Jerome I. Rotter, Eileen Crimmins, Pinchas Cohen

**Affiliations:** 1 Leonard Davis School of Gerontology, University of Southern California; Los Angeles 90089, CA, USA.; 2 Clayton Foundation Laboratories for Peptide Biology, Salk Institute for Biological Studies; La Jolla, CA 92037, USA.; 3 Institute of Computation, Altos Labs; San Diego 92114, CA USA.; 4 The Institute for Translational Genomics and Population Sciences, Department of Pediatrics, The Lundquist Institute for Biomedical Innovation at Harbor-UCLA Medical Center; Torrance, CA 90502, USA.; 5 Department of Biological Chemistry, David Geffen School of Medicine, University of California; Los Angeles, Los Angeles 90095, CA, USA.; 6 Department of Population and Public Health Sciences, Keck School of Medicine, University of Southern California, Los Angeles, CA 90032, USA.; 7 Department of Ophthalmology, Gavin Herbert Eye Institute, University of California Irvine; Irvine 92697, CA, USA.; 8 Graduate School of Medicine, Tohoku University, Sendai, Japan.; 9 Bursky School of Public Health, Washington University in St. Louis, MO 63130, USA.; 10 Department of Epidemiology and Environmental Health, School of Public Health and Health Professions, State University of New York at Buffalo, Buffalo, NY 14214, USA.

**Keywords:** mitochondria, microprotein, diabetes, obesity, therapeutic

## Abstract

**Rationale:**

Obesity and type 2 diabetes (T2D) are growing threats to human health, and their genetic basis is complex and not fully understood. Furthermore, the mitochondrial genome has been shown to encode for many microproteins that have a variety of biological effects. In this study we explore a newly discovered **m**itochondrial-**d**erived micro**p**rotein (MDP) that may be responsible for some forms of diabetes in humans.

**Methods:**

We have performed a mitochondrial genome wide interaction study (MiWIS) and discovered a SNP that lies within the gene for an MDP and is associated with type 2 diabetes. We then used cell culture to confirm that this MDP has biological activity and used mass spectrometry to detect it. This novel MDP and more potent analogues were then administered in murine, *in vivo* studies in models of diabetes and obesity to determine the effects. Further analysis of the *in vivo* studies was performed with transcriptomic and proteomic techniques.

**Results:**

Our MiWIS found a SNP associated with type 2 diabetes in 3 independent cohorts that is found within a novel MDP that we have called MENTSH (**M**DP **E**ncoded in the **N**D-**T**wo **S**ubunit of **H**umans). This common SNP is found in populations indigenous to the Americas that interrupts the start codon of MENTSH. Murine *in vivo* studies demonstrate that MENTSH administration improves insulin signaling, while analogues of MENTSH can potently block weight gain caused by a high fat diet. Mechanistically, our studies show that MENTSH activates AKT signaling in muscle, while reducing AKT signaling in fat.

**Conclusions:**

These observations highlight a new cause of metabolic dysfunction in a vulnerable population, suggesting that MENTSH could be an innovative, precision medicine approach to treating T2D.

## Introduction

Type 2 diabetes (T2D) and obesity are ever-increasing problems in the world, and type 2 diabetes is a major cause of morbidity and mortality in the Americas 1. In Mexico, T2D is responsible for at least one-third of all adult deaths 2, 3. In the USA, Mexican Americans are more than twice as likely to be diagnosed with T2D under the age of 40 compared to non-Hispanic Whites (NHW) (35% *vs.* 14.4% respectively), and this has been correlated with worse outcomes 4. In contrast to 17 other causes of mortality, the “diabetes and kidney disease” category is one of the two leading causes of death that is higher in the American Latino population compared to NHW 5. Furthermore, there exists racial and ethnic differences in response to T2D medicines such as metformin with most racial groups having a higher failure rate than NHW 6. Although socioeconomic status certainly plays a role in the higher incidence and severity of T2D in Mexican Americans, we hypothesized that mitochondrial genetics would also play a role in T2D in this population, in addition to the nuclear genome, which was previously implicated in T2D by several studies, as the mitochondrial DNA (mtDNA) is highly conserved within ethnicities due to the uniparental maternal inheritance and the lack of recombination 7-11.

An increasing number of microproteins (here defined as a protein with less than 100 amino acid residues) are being discovered and their function better defined. It has now been well established that the artificial cutoff of 100 amino acids for proteins is arbitrary, and more microproteins are being discovered in both the nuclear and mitochondrial genomes in multiple species by many labs 12-23. With this explosion in the number of functional proteins encoded by the genome comes an exponential increase in biological complexity as well as an opportunity to intervene in disease processes. Mitochondria are unique in that they have their own compact genome that classically encodes for 13 proteins, 22 tRNAs, and 2 rRNAs. Discoveries from our lab and others have expanded this list to include additional microproteins that we call **m**itochondrial **d**erived micro**p**roteins (MDPs) such as humanin, MOTS-c, SHMOOSE, the SHLPs, mtaltND4, and gau 16, 24-28. In this study we have identified a novel MDP using a genetics first approach and found that it plays a role in T2D. Mechanistically, we find that it differentially interacts with the AKT pathway in tissues and that its administration to cells can ameliorate the negative effects of genetic deficiency.

## Materials and Methods

### HRS Cohort

The sample was drawn from the *Health and Retirement Study* (HRS), a nationally representative sample of households of older Americans in the 48 contiguous United States. Participants consent to be interviewed biennially on a broad range of economic, psychological, physical, and biological health measures. For the present study, we included all participants who provided a sample for genotyping and a sample with which to characterize glycosylated hemoglobin (HbA1c) levels. The HbA1c assay was performed on dried blood samples (DBS) using a Bio-Rad Laboratories Variant II High Pressure Liquid Chromatography (HPLC) System (Hercules, CA) optimized to accommodate the limited microliter volume available from a DBS sample. Principal reagents were the U.S. Food and Drug Administration (FDA)-cleared Variant II Hemoglobin A1C Program (FDA K070452 and FDA K130860) obtained from Bio-Rad. DBS assay results were verified by comparing %HbA1c values obtained from 177 DBS samples versus DBS-matched blood samples (all samples analyzed on the Variant II). The correlation coefficient of the linear regression (LR) comparison was R^2^ = 0.99. Because HbA1c was not assayed in the 2016 venous blood collection but was one of the assays done using DBS collected in 2014 and 2016, HbA1c is based on the venous blood equivalent value associated with the DBS assay.

#### HRS GWAS Genotyping

Genotyping was performed by the National Institutes of Health (NIH) Center for Inherited Disease Research (CIDR; Johns Hopkins University, Baltimore, MD) using the Illumina Human Omni2.5-Quad BeadChip (Illumina, San Diego, CA), with coverage of nearly 2.5 million single nucleotide polymorphisms (SNPs). DNA samples for HRS participants used for this study were collected at several waves (2006-2010). HRS followed standard quality control recommendations to exclude samples and markers that obtained questionable data, including CIDR technical filters, removing SNPs that were duplicates, had missing call rates ≥ 2%, > 4 discordant calls, > 1 Mendelian error, deviations from Hardy-Weinberg equilibrium (at *p*-value < 10^-4^ in European samples), and sex differences in allelic frequency ≥ 0.2). All quality control checks and imputation procedures were implemented by the HRS 29.

#### MiWIS (Mitochondrial genome-Wide Interaction Study)

A mitochondrial genome-wide interaction study (MiWIS) was conducted to evaluate SNP-by-BMI interactions on hemoglobin A1C (HbA1c) levels. We analyzed data from Hispanic participants from the Health and Retirement Study (HRS) who had available genetic (2006-2010) 30 and biomarker data (2006-2016) 31. Genotype data and mitochondrial and nuclear principal components (mtPCs, nucPCs) were derived from previously processed and quality-controlled datasets 32. Analyses were restricted to individuals who self-identified as Hispanic. Covariates included age, sex, BMI, smoking status, and the top five nuclear and mitochondrial principal components to account for population substructure.

To isolate untreated glycemic variation, individuals reporting diabetes medication use, insulin therapy, or controlled diabetes were excluded, leaving approximately 918 individuals. MiWIS was performed using PLINK 2.0 33 with linear association tests modeling additive effects for SNP-by-BMI interactions (--glm interaction) on HbA1c levels. SNPs with a minor allele frequency (MAF) below 1% and individuals with call rates below 80% were excluded (--maf 0.01 --mind 0.2). A total of 90 mitochondrial SNPs were included in the analysis. Multiple test correction was completed using the Benjamini-Hochberg false discovery rate (FDR) procedure.

Following genome-wide interaction testing, the MENTSH SNP emerged as a top candidate. To further characterize this SNP, generalized linear models were used to assess its interaction with BMI on HbA1c. Model predictions were visualized across the BMI spectrum and stratified by MENTSH genotype.

#### Multi-Ethnic Study of Atherosclerosis (MESA) Cohort

The Multi-Ethnic Study of Atherosclerosis (MESA) is a study of the characteristics of subclinical cardiovascular disease and the risk factors that predict progression to clinically overt cardiovascular disease or progression of the subclinical disease 34. MESA consists of a diverse, population-based sample of an initial 6,814 men and women aged 45-84 without known cardiovascular disease. 38 percent of the recruited participants were self-described as White, 28 percent African American, 22 percent Hispanic, and 12 percent Chinese. Participants were recruited from six field centers across the United States: Wake Forest University, Columbia University, Johns Hopkins University, University of Minnesota, Northwestern University and University of California - Los Angeles. Participants are being followed for identification and characterization of cardiovascular disease events, including acute myocardial infarction and other forms of coronary heart disease (CHD), stroke, and congestive heart failure; for cardiovascular disease interventions; and for mortality. The first examination took place over two years, from July 2000 - July 2002. It was followed by six examination periods that were 17-20 months in length, including the recently completed Exam 7 (2022-2024). Participants have been contacted every 9 to 12 months throughout the study to assess and adjudicate clinical morbidity and mortality. Informed consent was obtained for extensive data sharing (dbGaP) and genetic/omic studies, including candidate genes (NHLBI CARe), genome-wide scans (NHLBI SHARe), exome sequencing (NHLBI ESP) and, most recently, the NHLBI TOPMed program.

#### GWAS Genotyping

All MESA participants were genotyped on the Affymetrix Genome-Wide Human SNP Array 6.0 (Affymetrix, Santa Clara, CA, USA) at the Affymetrix Research Services Lab. 6880 samples passed initial genotyping QC, including 1,303 Hispanic American samples used in this study. African American samples were genotyped at the Broad Institute of Harvard and MIT as part of the CARe project. Affymetrix performed wet lab hybridization assay, and plate-based genotype calling using Birdseed v2. Sample QC was based on call rates and contrast QC (cQC) statistics. Broad performed similar QC for the CARe sample. Additional sample and SNP QC were carried out at the University of Virginia, including sample call rate, sample cQC, and sample heterozygosity by race at the sample level; Outlier plates checking by call rate, median cQC or heterozygosity at plate level. Four samples were removed due to low call rate (<95%). Cryptic sample duplicates or unresolved cryptic duplicates were dropped. Unresolved gender mismatches were also dropped. At the SNP level, we excluded monomorphic SNPs across all samples; SNPs with missing Rate was > 5% or observed heterozygosity > 53% were also excluded.

#### Data Analysis

To confirm the ethnic specific SNP T4977C (rs28357981) association with HbA1c, we carried out association analysis in MESA Mexican American samples which was defined as self-reported Hispanics and sub-grouped into Mexican Americans via principle component analysis (PCA). Out of the 1303 self-reported Hispanic American subjects in MESA, we identified 868 as Mexican American group, and 435 as Caribbean subgroup via PCA analysis. Linear regression analysis was run as HbA1c = β0 + βage Age + βsex Sex + βBMI BMI + βPC1PC1 + βPC2PC2 + βG SNP, where SNP T4977C was modelled as risk allele (T) carrier versus wild type. A significant association between SNP T4977C and HbA1c was confirmed in MESA Mexican American samples with p-value = 0.0126.

### HTN-IR Cohort

The Hypertension-Insulin Resistance Family Study (HTN-IR) was designed to examine the genetic basis of hypertension and insulin resistance using a family-based design 35. Family members of Mexican American probands with documented hypertension were recruited from the Los Angeles area.

#### GWAS Genotyping

All samples were genotyped on the Illumina HumanOmniExpress BeadChip, and alleles were called using GenomeStudio software (Illumina, San Diego, CA) 36, 37. Samples with call rates > 0.98, single nucleotide polymorphisms (SNPs) with call rates > 0.99, and minor allele frequency (MAF) > 0.001 passed laboratory quality control, with 22,000 additional SNPs manually reviewed for clustering accuracy. Samples were removed from analysis if the overall call rate was < 0.98, if the samples were genetic outliers for sex and admixture proportions, if the samples were monomorphic, or if there was inconsistent fingerprinting from existing SNP data (PMCID: PMC4407862). The primary inferential SNPs did not exhibit differential missingness by trait, had a SNP call rate > 98%, and did not depart from Hardy- Weinberg equilibrium expectations. Pedigrees were examined for consistency of stated family structure. Each SNP was examined for Mendelian inconsistencies using PedCheck (Program for Detecting Marker Typing Incompatibilities in Pedigree Data, http://watson.hgen.pitt.edu/register/docs/pedcheck.html), and inconsistencies were converted to missing. Population substructure was estimated using ADMIXTURE version 1.21 (http://www.genetics.ucla.edu/software/admixture) based on SNPs that passed quality control. Data from the HapMap Project, including Mexican ancestry, were used as reference populations 38. Admixture proportions were included as covariates in the tests of association.

#### Data Analysis

Associations between SNP T4977C and DM were evaluated using the general estimating equations (GEE1) method to adjust for familial relationships in HTN-IR. To control for potential confounding effect, we adjust for age, sex, BMI, and the 3 admixture proportions generated from the admixture analysis, i.e. DM ~ age, sex, BMI, q1, q2, q4, and T4977C. There were 116 T allele carriers among 759 controls (15.3%), versus 28 out of 113 (24.8%) DM subjects who carried the T allele. Significant association between SNP T4977C and DM was found in HTN-IR sample with a p-value of 0.001.

### MENTSH Antibody

A custom MENTSH antibody was created by sending the sequence to Yenzym Antibodies (Brisbane, CA), where they created the antibody using rabbits as the host species. Because there is some controversy about the proper stop codons for mitochondrially translated proteins, we made antibodies targeting the larger protein sequence as follows: MKPNPATQNLSMLLNYPHRMNNSSSTVQP.

### MENTSH IP for Mass Spec

Approximately 1x10^7^ HEK293 cells were used for immunoprecipitation. Briefly, cells were lysed with Pierce RIPA lysis buffer (Thermo Fisher Scientific, Waltham, MA, USA) plus the Halt protease and phosphatase inhibitor cocktail (Thermo Fisher Scientific, Waltham, MA, USA). The lysate was incubated on ice for 10 minutes and then homogenized using a sonicator, and the supernatant was collected by centrifugation at 15,000 x g for 15 minutes at 4 °C. MENTSH was immunoprecipitated using Dynabeads Protein G (Thermo Fisher Scientific, Waltham, MA, USA) conjugated to 10 μg of a custom MENTSH antibody (Yenzym). Following MENTSH immunoprecipitation sample was eluted from beads using 50mM Glycine pH 2.8, and eluent was pH neutralized using Tris HCl pH 7.5. Complete eluent was then processed for protein identification.

### Mass Spec

#### Sample Preparation

Samples were mixed with equal volume of digestion buffer (8M Urea, 0.1M Tris-HCl pH 8.5) followed by reduction and alkylation via sequential 20-minute incubations with 5 mM TCEP and 10 mM iodoacetamide at room temperature in the dark while being mixed at 1200 rpm in an Eppendorf thermomixer. 20 μl of carboxylate-modified magnetic beads (CMMB and also widely known as SP3) was added to each sample followed by 100% acetonitrile to increase the final acetonitrile concentration to greater than 95% to induce peptide binding to CMMB. CMMB were washed 3 times with 100% acetonitrile and peptides were eluted with 50 μl of 2% DMSO. Eluted peptide samples were dried by vacuum centrifugation and reconstituted in 5% formic acid before LC-MS/MS analysis.

#### LC-MS Method

The Vanquish Neo LC system coupled to a timsTOF HT mass spectrometer (Bruker Daltonics) was used for DDA- PASEF data acquisition. Peptide samples were separated using Bruker PepSep C18 Columns (150 μm ID, 15 cm lengths, 1.5 μm particle size) using an increasing gradient of 5-10% mobile phase B (80% ACN with 0.1% FA (v/v)) at a flow rate of 600 nl/min from 0 - 2 minutes. The flow rate was then reduced to 300 nl/min flow rate to deliver a gradient of 10-10.5% B from 2 - 2.25 minutes, 10.5-16% B from 2.25 – 8 minutes, 16-30% B from 8-23 minutes, 30-45% B from 23 - 26 minutes, and 45-60% B from 26 to 27 minutes. The flow rate was then increased to 500 nl/min for the last step in which the gradient was increased to 95%B over 0.6 minutes and then held at 95%B until the 30 min gradient was completed.

The TIMS configuration utilized a ramp time of 100 ms covering the ion mobility range from 0.6 - 1.6 Vs/cm2 and an accumulation time of 50 ms, resulting in a total cycle time of 0.53 sec. Data acquisition was performed using a DDA-PASEF mode with 4 PASEF scans covering a mass range from 100 m/z to 1700 m/z with charge states set from 0 to 5+. The capillary voltage was set at 4500 V.

#### Data Analysis

The acquisition data searching was conducted using FragPipe (version 1.6.10.43) against the human reference proteome from Uniprot with the microprotein amino acid sequence appended and no enzyme specificity considered.

### MTT

The MTT (MTT, 3-(4,5-Dimethyl-2-thiazolyl)-2,5-diphenyl-2H-tetrazolium Bromide, Thiazole Blue) assay is a readout of a number of possible biological functions (e.g. cell division, mitochondrial activity, cell death). It is a membrane permeable dye that changes from yellow to blue when reduced. In cells this can be caused by many factors but is thought to mainly be from an NADH/NADPH dependent mechanism 39. For our screen we seeded 96-well plates with 10,000 cells/well of 3T3-L1 cells (ATCC, Manassas, VA, USA) and grew them overnight in 10% FBS DMEM. Cells were then treated with either 100uM of MENTSH/analogue or PBS overnight (n = 16 wells/group). MTT solution was added to each well for a final concentration of 0.5 mg/mL for 1-2 hours. MTT lysis buffer (50% dimethylformamide (DMF), 15% sodium dodecyl sulfate (SDS), ~5% Acetic Acid to pH 4.7) was added, and the plates were incubated at 37 ºC overnight. Plates were read at 570 nm. On each plate we had a negative control (PBS) as well as MENTSH positive control. Plates were normalized to the positive control of each plate.

### Pharmacokinetic Study

Pharmacokinetic studies were performed in 15-week-old C57BL/6J male mice. Mice were injected IP with 2.5 mg/kg of MENTSH and blood collected into EDTA tubes at time 0, 15 minutes, 30 minutes, 60 minutes, 180 minutes, 360 minutes, and 24 hours (N = 5 at each time point). The blood was centrifuges for 15 minutes at 1500 rcf, and plasma was collected and analyzed by us in house MENTSH ELISA. Half-life was calculated fitting the data to a non-linear, one-phase decay model using GraphPad Prism.

### Mouse Experiments

All animal experiments have been approved by the USC IACUC committee under protocol numbers 11926 and 11927.

#### Normal Chow Experiment

12-week-old C57BL/6J male mice were used (N = 14 and 8 for Control and MENTSH group respectively). Mice were multiply housed with 4-5 individuals per cage. Mice were injected with 2.5 mg/kg of MENTSH or PBS twice a day. Bodyweights and food consumed was measured approximately every other day.

#### HFD Experiment

10-week-old C57BL/6J male mice (N = 15/group) were placed on a high fat diet (60% fat, Research Diets D12492). They were IP injected with 2.5 mg/kg of MENTSH or PBS twice a day, or injected with 5 mg/kg of MENTSH once a day. Body weights and food consumed were measured approximately every other day. On day 10 of treatment, mice were fasted overnight and a GTT performed.

#### GTT

Mice were fasted overnight and injected IP with 2 g/kg of glucose the following morning. Blood glucose levels were measured at 0, 15, 30, 60, and 120 minutes using blood collected from the tail and measured by using a glucometer (Freestyle Lite Blood Glucose Monitoring System, Abbott, Chicago, IL, USA).

#### Long-term HFD Mouse Experiment

Starting at 6 weeks of age, female C57BL/6J mice were placed on a high fat diet (60% fat, Research Diets D12492). After 4 months, mice were injected with either PBS, MENTSH, or MENTSH analogue M32. On day 51 of treatment, a GTT was performed and a week later, an insulin tolerance test (ITT). Body composition was performed as previously describes 40. In short, animals were measured on days 3, 25, 39, 46, 61 using an NMR-based analyzer (Bruker Mini-Spec LF90II). Mouse weights and food consumption was measured approximately every other day.

#### Insulin Tolerance Test (ITT)

Mice were faster for 4-6 hours and then IP injected with 0.75 U/kg of insulin on day 58 of treatment. Blood glucose was measured from the tail using a glucometer (Freestyle Lite Blood Glucose Monitoring System, Abbott, Chicago, IL, USA) at time 0, 15 minutes, 30 minutes, 45 minutes, 60 minutes, and 120 minutes.

#### DIO Experiment

16-week-old, male, DIO C57BL/6J mice were obtained from Jackson Laboratory (Bar Harbor, ME, USA). These mice had been started on a HFD starting at 6 weeks of age. After 2 weeks of acclimation in our facilities, the mice were injected with either PBS, MENTSH, or MENTSH analogue M32-PH. Body composition was measured as above, and mouse weights and food consumption was measured approximately every other day.

#### Ob/ob Mice Experiment

5-week-old, male, ob/ob (B6.Cg-Lep) mice were purchased from Jackson Laboratories (Strain 000632, Jackson Laboratories, Bar Harbor, ME, USA) and allowed to acclimate for 3 weeks. Starting at 8 weeks of age, mice were injected IP twice a day with PBS or MENTSH analogue M32-PH (2.5 mg/kg). On day 8 of treatment, 4 mice from each group were placed in a metabolic cage (see below) for 5 days.

Body composition was measured as above, and mouse weights and food consumption were measured approximately every other day. GH and insulin measurements were measured by MSD following the company’s instructions.

#### Metabolic Cages

Metabolic cages experiments were performed by the Aging Murine Phenotyping Core at USC Leonard Davis School of Gerontology. Metabolic profile was obtained using the TSE PhenoMaster system (TSE Systems, Chesterfield, MO, USA) that measured food and water intake, energy expenditure, and locomotor activity.

#### Metabolomics

17-week-old C57BL/6J, male DIO mice were purchased from Jackson Laboratories. Metabolomics on plasma was performed as previously described 41. DIO mice were treated with MENTSH or PBS for 3 days before sacrifice and plasma collected (N = 6 mice/group). Plasma was sent to Metabolon (Durham, NC, USA). Analysis of the data was conducted using Ingenuity Pathway Analysis (Qiagen, USA).

#### Pump Studies

14-week-old male C57Bl6/J mice were implanted with osmotic pumps (Alzet, Cupertino, CA, USA) subcutaneously between the scapula. The pumps were filled with PBS, MENTSH (5 mg/kg/day) or M32PH (2.5 mg/kg/day). The mice and the food were weighed every weekday.

#### Statistics

Data plotted as a bar graph was analyzed using ANOVA followed by a Fisher’s LDS test using Graphpad Prism. Longitudinal studies were analyzed using a t-test comparing the experimental group to the control group to determine significance. Mice were only censored if they were determined to be sick, injured, or on recommendation by the university veterinarians.

### Cell Culture

All cells were incubated at 37 ºC in a humidified atmosphere with 5% CO_2_ in air. Mouse C2C12 myoblasts (American Type Culture Collection, ATCC, Manassas, VA, USA) were cultured in high glucose containing Dulbecco’s Modified Eagle growth medium (GM) (DMEM, Thermo Fisher Scientific, Waltham, MA, USA) supplemented with 10% (v/v) fetal bovine serum (FBS, Omega Scientific, Tarzana, CA, USA). ARPE-19 transmitochondrial cybrid cell lines were generously provided by Dr. Kenney. ARPE-19 cells were cultured in Dulbecco’s modified Eagles medium and Ham’s F12 medium with HEPES buffer (DMEM/F12, Thermo Fisher Scientific, Waltham, MA, USA) containing 10% fetal bovine serum, 0.5 mM sodium pyruvate (Thermo Fisher Scientific, Waltham, MA, USA) and 1200 mg/L sodium bicarbonate (Thermo Fisher Scientific, Waltham, MA, USA).

### Seahorse FAO Assay

The Seahorse XF Palmitate-BSA FAO substrate kit (Agilent/Seahorse Bioscience, Santa Clara, CA) was used to assay fatty acid oxidation in, ARPE-19 transmitochondrial cybrid cells, according to the manufacturer’s instructions. Briefly, ARPE-19 transmitochondrial cybrid cells were grown as before. The day before the assay, the cells were placed in substrate limited medium (DMEM without glucose, glutamine, or phenol red supplemented with 0.5 mM Glucose, 1.0 mM GlutaMAX, 0.5 mM Carnitine and 1% FBS). At the beginning of the second day of the experiment, the number of cells and cell density were ensured to be at least 90% with cells in monolayers and evenly distributed. 45 minutes prior to assaying the cells, the substrate limited medium was exchanged for fatty acid oxidation assay buffer (111 mM NaCL, 4.7 mM KCl, 1.25 mM CaCl_2_, 2.0 MgSO_4_, 1.2 mM NaH_2_PO_4_, 2.5 mM glucose, 0.5 mM carnitine, and 5 mM HEPES pH 7.4). During instrument calibration (30 minutes), the cells were switched to a CO_2_-free, 37 ºC, incubator. At the start of the assay Palmitate:BSA or BSA control was added and cellular consumption rates (OCR) were measured under basal conditions using the Seahorse XF96 analyzer (Agilent/Seahorse Bioscience, Santa Clara, CA). The data were then analyzed after normalization.

### Western Blot Analysis

C2C12 myoblasts were seeded on 6-well plates (Nunclon™ Delta; Thermo Fisher Scientific, Waltham, MA, USA). When the myoblasts reached 95-100% confluence, the cells were rinsed with Dulbecco’s Phosphate-Buffered Saline (DPBS, Lonza Bioscience, Walkersville, MD, USA) and the GM was replaced by differentiation medium (DM) containing high glucose DMEM, 2% (v/v) horse serum (HS, Thermo Fisher Scientific, Waltham, MA, USA) to promote fusion into myotubes. Fresh DM was changed every second day. On the sixth day DM was replaced with serum free DM media supplemented with either water, MENTSH (100 uM) (GenScript Biotech, Piscataway, NJ, USA), or M32 (100 uM) (GenScript, Biotech, Piscataway, NJ, USA). The following day cells were treated with 10 nM bovine insulin (Sigma-Aldrich, St. Louis, MO, USA) for 15 minutes. Cells were lysed with Pierce® RIPA lysis buffer (Thermo Fisher Scientific, Waltham, MA, USA) plus the Halt protease and phosphatase inhibitor cocktail (Thermo Fisher Scientific, Waltham, MA, USA). The lysates were incubated on ice for 10 minutes and then homogenized using a sonicator, and the supernatant was collected by centrifugation at 15,000 x g for 15 minutes at 4 ºC. Protein content in the cellular lysates were quantified using the Pierce® BCA protein Assay Kit (Thermo Fisher Scientific, Waltham, MA, USA). A total of 50ug protein was separated on a 4-20% Mini-Protean® TGX™ SDS-PAGE gels (Bio-Rad Laboratories, Hercules, CA, USA) and blotted onto PVDF membranes (Bio-Rad Laboratories, Hercules, CA, USA). Membranes were incubated with primary antibody at 4 ºC overnight according to the manufacturer’s instruction. After three washes with TRIS-buffered saline (Thermo Fisher Scientific, Waltham, MA, USA) containing 0.1% TWEEN-20 (Sigma-Aldrich, St. Louis, MO, USA) membranes were incubated at room temperature for 1 hour with the appropriate HRP-conjugated secondary antibody. Clarity Max™ Western ECL substrate (Bio-Rad Laboratories, Hercules, CA, USA) was used for detecting specific bands. Membranes were imaged on a Bio-Rad ChemiDoc XRS+ imager. Relative intensities of the bands in each condition were measured using Image J, a free software program provided by the National Institute of Health (Bethesda, Maryland, USA).

### MSD Measurement of AKT Phosphorylation

For mouse adipose and muscle tissues, Total and phosphorylated Akt (Ser473 and Thr308) were quantified using Meso Scale Discovery (MSD) electrochemiluminescence (ECL) whole-cell lysate assays. Briefly, tissue lysates were loaded onto MSD plates and incubated according to the manufacturer’s instructions. Electrochemiluminescent signals were captured and analyzed using a Meso QuickPlex SQ 120 plate reader (MSD).

### Reagents

The following antibodies were used in this study: Phospho-Akt (Ser473) (1:1000) (4060S, Cell Signaling Technology, Danvers, MA, USA), Phospho-Akt (Thr308) (1:1000) (13038S, Cell Signaling Technology, Danvers, MA, USA), Total Akt (4691S, Cell Signaling Technology, Danvers, MA, USA), Phospho-PTEN (Ser380) (1:1000) (9551S, Cell Signaling Technology, Danvers, MA, USA), Phospho-IRS-1 (Ser1101) (1:1000) (2385S Cell Signaling Technology, Danvers, MA, USA), GAPDH (1:1000) (5174S,Cell Signaling Technology, Danvers, MA, USA).

### Acute Protein and RNAseq experiments

12-week old, male C57BL/6J mice were fasted overnight and then injected with MENTSH (5 mg/kg) or PBS. 15 minutes after the first injection mice were injected with insulin (0.5 U/kg). 30 minutes after the first injection animals were sacrificed. For RNA seq experiments animals were sacrificed 2 hours after the first injection.

### Phospho-Mass Spec

Tissues were flash frozen using dry ice and samples were sent to Creative Proteomics (Shirley, NY, USA) for further analysis. Samples were trypsin digested, enriched with iron-FEMAC beads, and ran on their nano-LC-MS/MS platform.

### RNAseq

Total RNA was extracted from muscle, fat (inguinal white adipose tissue), and liver using the Direct-zol™ RNA MiniPrep (Zymo Research, Irvine, CA, USA), The protocol was performed according to the manufacturer’s instructions and RNA was measured at 260nm with the Nanodrop system (Thermo Fisher Scientific, Waltham, MA, USA). RNA purity and quality were evaluated by 260/280 and 260/230 ratios. RNA library preparation (mRNA-Seq Nu Quant) was done to enrich poly-adenylated RNA. Samples were sequenced on an Illumina NextSeq 550 platform for 75 single end cycles, quality ensured using FastQC, and mapped to the mouse reference genome (GRCm39) using kallisto as previously described 27. Normalized fold changes were used to estimate differential gene expression from among conditions by using the *DESeq2* package in R. The *DESeq2* PCA function was used to assess outliers and clustering of conditions in reduced dimensionality. *WikiPathway* enrichment was carried out on significantly different gene (FDR < 0.2) using the *clusterProfiler* package in R. The specific question was which terms differential expression of genes enriched against a background of expressed genes by these studied mice, which was accomplished using the enrichWP function. Genes within significantly enriched terms were extracted and plotted using custom scripts in R.

## Results

### Discovery of a SNP and Mitochondrial Microprotein Associated with Type 2 Diabetes

**Figure 1:**
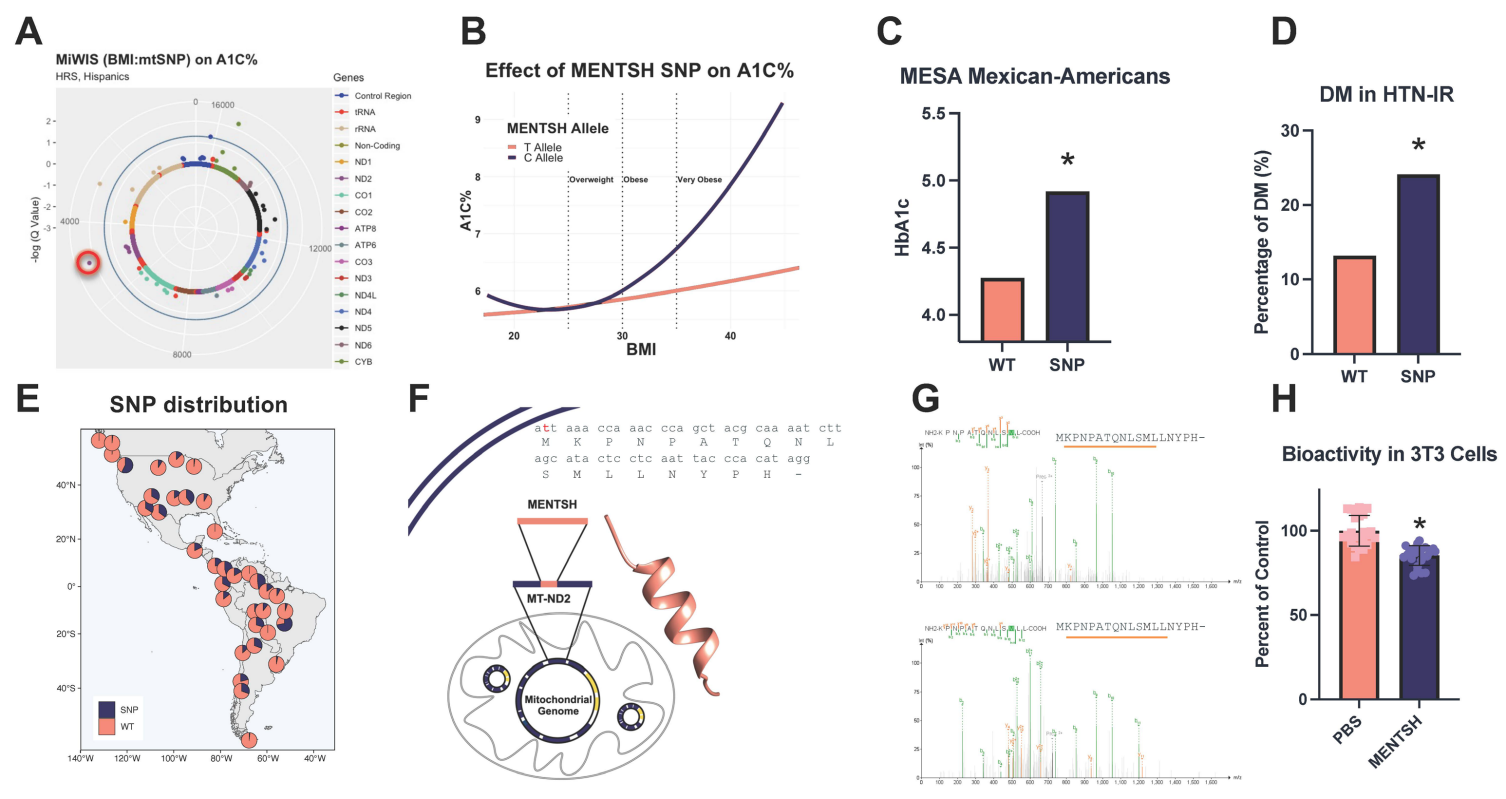
** A mitochondrial SNP is associated with an increased risk of type 2 diabetes (T2D) in overweight and obese individuals of indigenous American descent and codes for a functional microprotein.** (**A**) A mitochondrial wide interaction study (MiWIS) conducted on the Health and Retirement Study (HRS) cohort found that the MENTSH SNP was the most significant mitochondrial SNP that affected A1C% when interacted with BMI (q = .0055) (**B**) In the HRS, this SNP was associated with an increase in Hba1c levels dependent on BMI (p < 0.0005). (**C, D**) In two additional, independent cohorts (MESA & HTN-IR) we further confirmed the association of the SNP and HbA1c or T2D respectively (p = .0126, p = .0011 analyzed by a linear regression model and a general estimating equations method respectively). (**E**) When examining the distribution of this SNP in the world, we found it to be primarily in those of indigenous American descent. (**F**) The SNP is a synonymous mutation (highlighted in red) in the MT-ND2 gene and interrupts the start codon of a novel MDP we named MENTSH with a theoretical structure as shown. (**G**) We were able to detect several fragments of this microprotein using MS/MS and (**H**) it had biological activity in a cell culture assay in 3T3-L1 (mouse pre-adipocytes) cells (n = 16/group, p = .0002). Error bars represent s.e.m. *P < .05.

To discover additional MDPs, we used a mitochondrial-focused, genome-wide, interaction study (MiWIS) in the Health and Retirement Study (HRS), a population-representative study of US adults 50 years of age or older (n = ~15,000) 42. Testing the interactions between BMI and mtSNPs, we discovered an ethnic specific SNP T4977C (rs28357981) that was the most significant SNP associated with hemoglobin A1C levels in this older population, indicating an overall increase in blood glucose levels and likely T2D (Figure 1A). Further analysis of this SNP and HbA1C levels found that this SNP only increased A1C levels in those individuals that were overweight (BMI > 25) or obese (BMI > 30) (Figure 1B). Looking to replicate our findings in additional cohorts that had this SNP, we employed our analysis in both the Multi-Ethnic Study of Atherosclerosis (MESA) (n = 868) and Hypertension-Insulin Resistance (HTN-IR) cohorts 34, 35. In these independent and younger cohorts, we replicated this association in both the MESA and HTN-IR studies (Figure 1C and 1D). When we examined the frequency of the SNP by ethnicity, we found that it was primarily found in people of Native American descent and was found in approximately 20% of Mexican and Mexican Americans (Figure 1E) 43. As this SNP is a synonymous mutation with regards to the larger *MT-ND2* gene, we looked for an alternative explanation for this association and found that the SNP interrupts the start codon of an MDP that we named MENTSH (**M**DP **E**ncoded in the **N**D-**T**wo **S**ubunit of **H**umans) (Figure 1F). Using AlphaFold2 to model the structure of MENTSH, it is primarily an alpha-helix flanked by two unstructured regions (Figure 1F) 44. This start codon (ATT) is a mitochondrial specific start codon and is the same one that is used for the larger, encompassing *MT-ND2* gene. This SNP variant would lead to MENTSH not being produced by carriers. Using a custom antibody to pull-down MENTSH, we performed mass spectrometry and were able to verify the existence of MENTSH by detecting two different fragments (Figure 1G). When administered to 3T3-L1 mouse pre-adipocyte cells, MENTSH (100 µM) decreased metabolism as measured by MTT assay (Figure 1H).

### MENTSH and Its Analogues Improve Glucose Tolerance in Murine Models of Type 2 Diabetes

**Figure 2:**
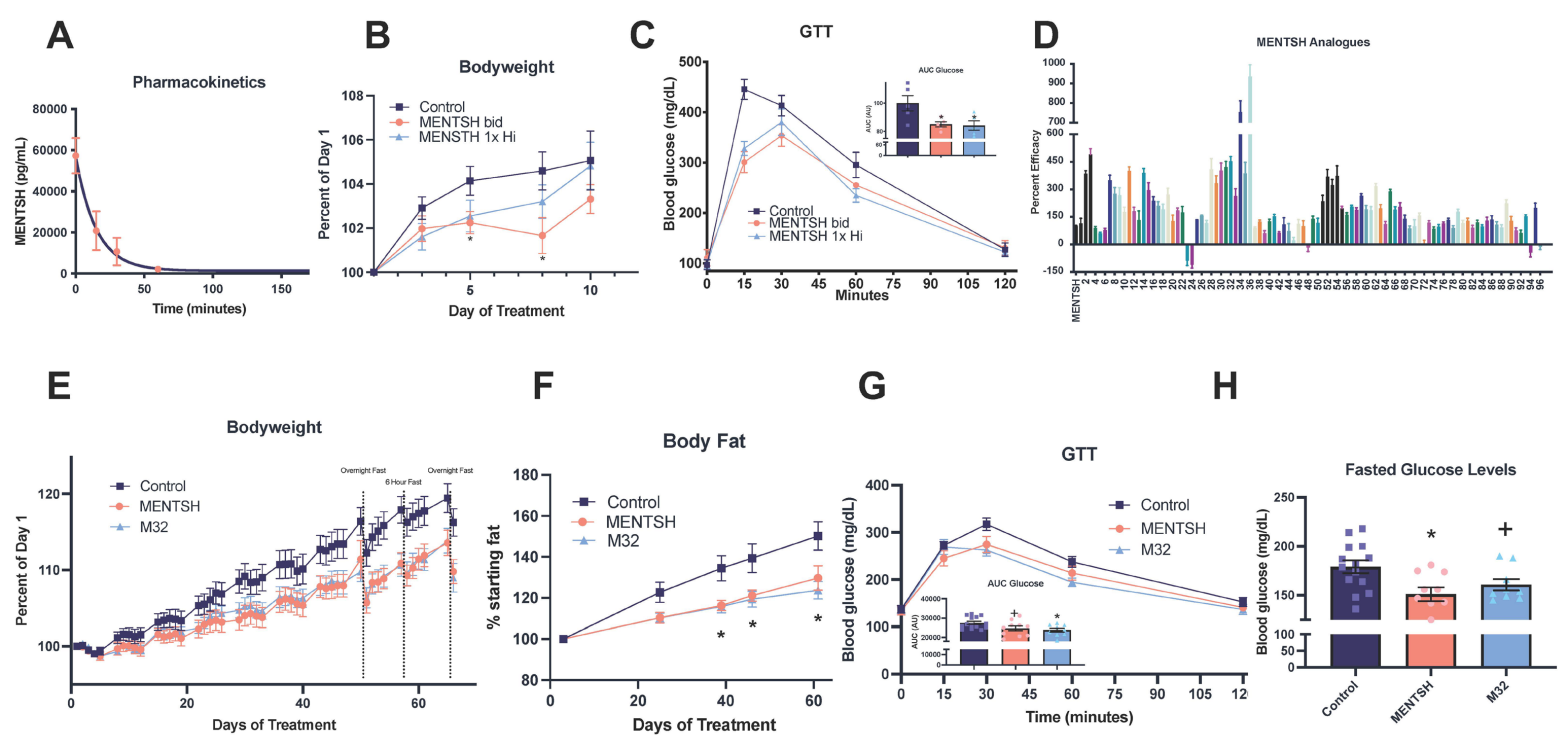
** MENTSH and its analogue improves insulin sensitivity in high-fat diet fed mice.** (**A**) Pharmacokinetic studies in mice find that MENTSH, similar to other MDPs, has a short half-life of only 10.4 minutes (N = 5/time point). Similar to the data in humans, MENTSH only has an effect in mice on a high fat diet where (**B**) a single high dose (20 mg/kg) trended to improved weight gain while a bid/twice a day low dose (2.5 mg/kg/injection bid) reduced body weight gain (N = 15/group, p = .0361 on day 5 and p = .021 on day 8). (**C**) A glucose tolerance test indicated a significantly improved glucose response in treated animals (N = 5/group, p = .0311 for bid, and p = .0237 for 1x Hi). (**D**) Alanine scanning and amino acid substitutions of the peptide using a broad screen for biological activity (MTT) indicate important sites of activity. (**E**) Both MENTSH and M32 significantly attenuated female mice weight gain on a HFD (N = 14, N = 9, N = 10 for control, MENTSH, and M32 groups respectively, p < .05 by day 25 onwards). (**F**) Body fat was reduced in the treated mice (p < .05 starting day 40). (**G**) GTTs were performed and MENTSH mice trended towards significance, while M32 treated mice had a significant improvement in AUC (p = .0900 and p = .0244 for MENTSH and M32 groups respectively). (**H**) 3-6 hour fasted blood glucose levels were significantly lower in the MENTSH treated mice and trended towards a decrease in M32 treated mice (p = .0084 and p = .1093 for MENTSH and M32 respectively). Error bars represent s.e.m. *p < .05, +p 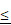.1.

To determine the *in vivo* effects of this microprotein we first injected mice intraperitoneally (IP) (2.5 mg/kg) with MENTSH to determine the pharmacokinetics and found that, like other smaller MDPs, the half-life of MENTSH is relatively short, at 10.4 minutes (Figure 2A) 45, 46. Following a similar protocol that we have previously used for MDP administration 25, 47, we injected 2.5 mg/kg twice a day (bid) IP into mice for 10 days, and found no changes in bodyweight, food consumption or blood glucose (Figure S2A-C), consistent with the human data showing no effect of this microprotein in normal weight individuals. In contrast, when placed on a high-fat diet (60% Fat, Research Diets D12492) and injected with either PBS, MENTSH bid (2.5 mg/kg/injection), or MENTSH once a day at a high dose (20 mg/kg), the treated mice trended towards less weight gain (Figure 2B), ate the same amount of food (Figure S2D), but responded significantly better to a glucose tolerance test (Figure 2C and quantified in the inset). Because of the small size of the microprotein, we performed an alanine scan to try to determine important amino acid residues as well as swapping in different amino acid residues to look at structure and function (Figure 2D and Table S1). In total, we screened 96 analogues and identified some that displayed increased efficacy and others with decreased efficacy in our assay. In general, we found that the N-terminal region was important for biological activity and that changes in overall alpha-helix propensity or hydrophobicity did not make a consistent difference (Figure 2D). To examine the effects of MENTSH over a longer treatment time, we placed mice on an HFD and treated them with either PBS, MENTSH (2.5 mg/kg bid), or a MENTSH analogue (M32 that we found to be more potent in our *in vitro* screen) (2.5 mg/kg bid IP) for over two months. The M32 analogue substitutes two aspartic acid residues in place of serine-11 and tyrosine-16 (Table S1). The treated mice trended towards a difference in bodyweight after 1 week of treatment and were significantly different by day 50 (Figure 2E) without a significant difference in total food intake (Figure S2E). Body fat percent of treated mice as measured by NMR trended towards a difference by day 25 and was significantly lower than control by day 39 (Figure 2F). Although lean mass percent was decreased by a high fat diet, M32 treatment significantly increased lean mass percent while MENTSH treatment did not (Figure S2F). GTT was conducted on day 51, with M32 showing a significant difference compared to control, while MENTSH treatment was trending towards a difference (Figure 2G). An ITT performed a week later (day 58) found that the initial blood glucose levels were significantly lower in the MENTSH-treated group and trended towards significance in the M32-treated group (Figure S2G). The AUC was not significantly different (Figure S2G and C).

**Figure 3:**
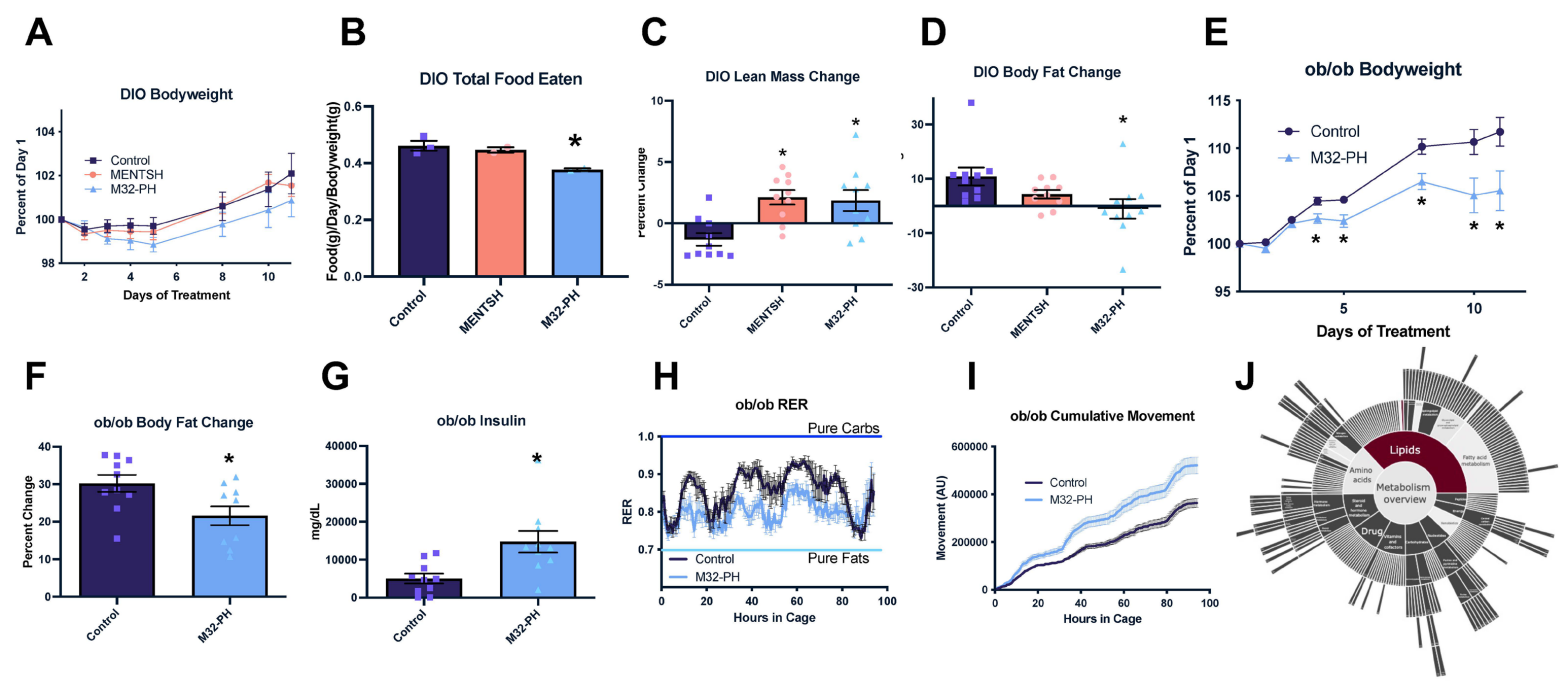
** MENTSH and its analogues improve metabolic fitness in additional type 2 diabetes models.** In a short-term study, (**A**) male DIO mice did not have a significant change in body weight (**B**) or food consumption for the MENTSH treated group, but the M32-PH group did have a significant decrease in total food consumption (p = .0262). Both the MENTSH and M32-PH treated groups (**C**) did have a significant increase in lean body mass (p = .0022 and p = .0045) and (**D**) M32-PH treated mice had a significant decrease in body fat (p = .0151). (**E**) In ob/ob mice, administration of M32-PH was able to reduce weight gain (p < .05 starting day 4). These mice also had a (**F**) decrease in fat mass (p = .0195) and (**G**) insulin levels were significantly increased (p = .0089). (**H**) RER was significantly reduced in treated mice (p < .0001) and (**I**) spontaneous movement was increased (p < .0001). (**J**) Metabolomic studies of DIO mice treated with MENTSH for 72 hours support the changes in RER with the category of fatty acid synthesis having the largest change in these mice. Error bars represent s.e.m.

While in the previous experiments treatment was initiated concurrent with the HFD, we wanted to examine the effects of MENTSH or another analogue (M32-PH, a shorter version of M32 that lacks the last two amino acids (Table S1)) on mice that were already obese. 16-week-old mice that had been on an HFD starting at 4 weeks of age were purchased from Jackson Labs and treated with either PBS, MENTSH, or M32-PH. Although there was no significant difference in body weight change over 2 weeks (Figure 3A), there was a significant change in total food eaten in the M32-PH group (Figure 3B) and a significant change in body composition as measured by NMR (Figure 3C, D). The MENTSH and M32-PH groups had significant increases in lean mass (Figure 3C) and the M32-PH group also had a significant reduction in the gain of body fat (Figure 3D). Examining another model of T2D we administered M32-PH to 7-week-old ob/ob mice, which have a mutation in their leptin gene, and found a significant decrease in weight gain (Figure 3E) without any change in food intake (Figure S3A). The treated mice also gained significantly less fat (Figure 3F) with no difference in lean mass (Figure S3B). Insulin levels were significantly increased (Figure 3G), while growth hormone levels trended towards an increase (Figure S3C). During this experiment, 4 mice from each group were placed in metabolic cages on day 8 of treatment. M32-PH treatment clearly shifted the respiratory exchange ratio (RER) towards fat metabolism compared to control (Figure 3H). The M32-PH group also had a significant increase in spontaneous movement (Figure 3I). To test alternative administration routes, we administered M32-PH or vehicle subcutaneously via implanted Alzet pumps for 2 weeks and examined body weight gain of the mice on an HFD. Similar to the IP-injected mice, we saw an attenuation in the weight gain of treated mice (Figure S3D). Because our data suggested that M32-PH shifts metabolism towards fat utilization, we used metabolomics to examine the plasma of 12-week-old DIO mice treated with MENTSH for 72 h. A partial least squares discriminant analysis found that the groups were well separated (Figure S3F). Supporting our data in ob/ob mice, there was a clear increase in fatty-acid metabolites, with the lipids category the only significantly changed category (Figure 3J and Table S2).

### MENTSH Differentially Affects AKT Signaling in Muscle and Fat

**Figure 4:**
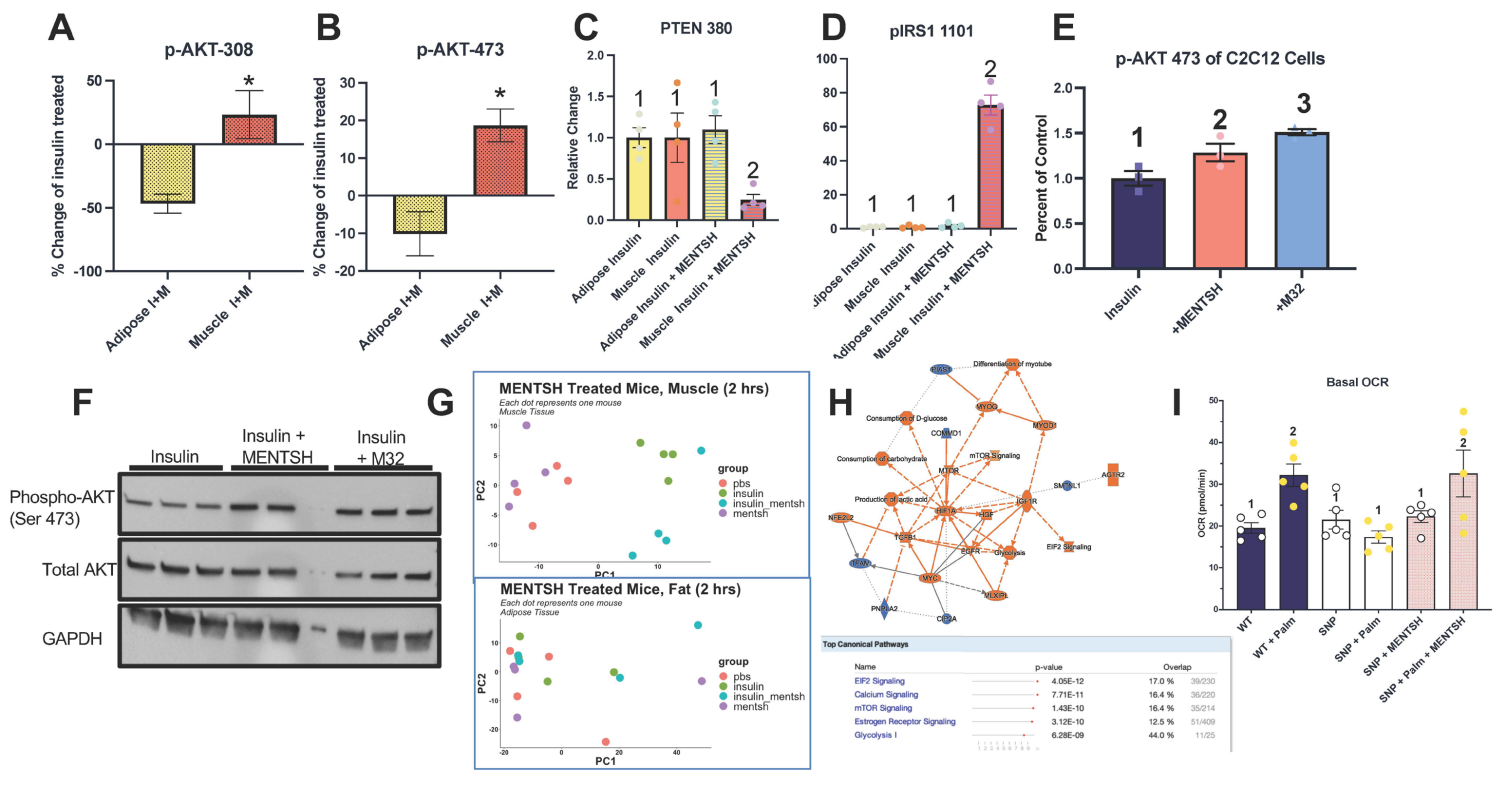
** MENTSH differentially attenuates AKT signaling in muscle and fat and can restore defects in fatty acid oxidation in SNP carrier cells.** Mice acutely treated with MENTSH and insulin have different AKT phosphorylation status. Treated mice have a decrease in AKT-308 and AKT-473 phosphorylation in the (**A**) fat (p = .0044) but an increase in (**B**) the muscle (p = .0010). (**C**) PTEN-308 phosphorylation was only decreased in the muscle of insulin treated mice (columns with different numbers are significantly different from each other p < .05) and (**D**) IRS1-1101 phosphorylation was increased (p < .0001) (columns with different numbers are significantly different from each other p < .05). (**E, F**) In differentiated C2C12 cells, we find that AKT phosphorylation was increased by both MENTSH and M32 treatment (columns with different numbers are significantly different from each other p < .05). (**G**) RNAseq of acutely treated mice further support the differential effect in muscle and fat as shown in the PCA plots (G upper and lower panel). N = 4/group. (**H**) IPA pathway analysis of the insulin +/- MENTSH muscle samples indicated that some of the top pathways were metabolism related. (**I**) A Seahorse fatty acid oxidation test was performed on RPE cybrid cells that were WT or SNP carriers. SNP carriers were deficient in fatty acid oxidation when palmitate was added to the media, and this was reversed with treatment of MENTSH (columns with different numbers are significantly different from each other). Error bars represent s.e.m.

To begin to dissect its mechanism of action, we examined the acute effects of MENTSH treatment in mice. Mice were injected with MENTSH or PBS with or without insulin. 15 minutes after injection, the mice were sacrificed, and tissues were collected and frozen on dry ice. Testing AKT phosphorylation at the 308 site (Figure 4A) and 473 site (Figure 4B) by MSD, we saw a differential effect in the muscle and adipose tissue of mice treated with insulin by WB. In muscle, AKT phosphorylation was increased at both sites, while it was decreased in adipose tissue (Figure 4A, B and Figure S4A). Further examining the effects on AKT signaling, we looked at both the PTEN-380 and IRS1-1101 phosphorylation sites and observed that they were decreased and increased, respectively, only in the muscle of insulin + MENTSH-treated mice, (Figure 4C, D and S4B). Replicating this finding in C2C12 murine muscle cells, we found that both MENTSH and M32 increased AKT signaling, as measured at the AKT-473 site (Figure 4E). To examine the transcriptional changes that occur with MENTSH treatment, mice were injected with either PBS or MENTSH (5 mg/kg), and then tissues were collected after 2 hours. Corroborating our protein findings, PCA analysis of the RNAseq data showed a distinct clustering of MENTSH + insulin *vs.* insulin treatment alone in the muscle (Figure 4G bottom), while there was no distinct clustering in adipose tissue (Figure 4G top). In the liver, we observed a distinction between insulin and non-insulin treated tissue but no difference between MENTSH or non-MENTSH treated mice (Figure S5A). Further analysis of the RNAseq data using IPA software (Qiagen, Redwood City, CA, USA) indicated that the mTOR signaling and glycolysis signaling pathways were major targets of MENTSH treatment in the muscle (Figure 4H and S5C). Additionally, EIF2, calcium, and estrogen signaling were notable as well. Looking at an earlier time point, we used phospho-mass spec to examine muscle and adipose tissue changes after 15 minutes of MENTSH treatment. Overlapping with the RNAseq data and western blots, pathways implicating the mTOR pathway, glycolysis, and insulin signaling were enriched in the muscle and adipose tissue (Figure S5C & S5D). Because of the difficulty in genetically manipulating the mitochondrial genome in cells, we turned to a human cybrid cell line model where the cell lines all carry the same nuclear genome but have mitochondria from either SNP carriers or non-carriers. Because our data suggested that MENTSH altered fatty acid utilization, we performed a fatty-acid oxidation assay and measured the oxygen consumption rate (OCR) using a Seahorse XF Analyzer (Agilent, Santa Clara, CA, USA) (Figure 4I). While the OCR of WT or SNP carriers were the same at baseline (BSA), SNP carriers failed to increase OCR when palmitate was added to the media. This lack of response to palmitate was reversed when the SNP carriers were treated with MENTSH for 24 hours before palmitate addition (Figure 4I).

## Discussion

Although nuclearly encoded proteins have been implicated in T2D 7-10, 48, 49, in this study we discovered the first mitochondrial SNP that is associated with an increased risk of T2D in this population and have replicated this finding in an additional two independent cohorts. Because of the uniqueness of mitochondrial genetics and its uniparental inheritance, this SNP is primarily found in populations of indigenous American descent. Further investigation found that this mutation seems to only affect individuals who are overweight or obese and may be another example of a “thrifty gene” that was beneficial during pre-modern times that only became detrimental in the modern era. We replicated this finding in our mouse studies where MENTSH only had effects in mice that were obese.

Because this SNP is a synonymous mutation with regards to the larger, encompassing, *MT-ND2* gene, we looked for alternative explanations for this association and discovered that this genetic variation interrupts the start codon of the novel, small open reading frame that encodes the mitochondrial microprotein we call MENTSH. This is similar to previous studies in both the nuclear and mitochondrial genomes that have found several other physiologically-relevant microproteins that are encoded by alternative, small open reading frames within larger genes 26, 27, 50. Although carriers should theoretically not produce MENTSH, future studies will need to confirm the absence of MENTSH in SNP carriers.

Looking at the mechanism of action of MENTSH, our metabolic cage data and metabolomics data suggested that treated mice switched fuel utilization to fat, and we found that MENTSH differentially affects AKT phosphorylation in the muscle and fat. Further RNAseq and phospho-mass spec experiments both identified glycolysis and the insulin/IGF/mTOR signaling pathways as immediate effectors of MENTSH treatment. Interestingly, EIF2, calcium, and estrogen receptor signaling were also changed in MENTSH treated muscle. These three pathways have previously been implicated in both metabolism and diabetes, and future studies will be needed to determine their role in MENTSH signaling 51-53. Using a human cybrid cell line that had the MENTSH SNP, we found that SNP carriers had a deficiency in FAO response and that this could be rescued by MENTSH treatment, suggesting that MENTSH administration may be a viable treatment option for MENTSH deficient people.

Additionally, we have identified two analogues (M32 and M32-PH) that have an improved efficacy compared to MENTSH. These analogues replace two amino acids that could theoretically be phosphorylated (serine-11 and tyrosine-16) with a negatively charged aspartic acid residue. While we think that the mechanism of action of these analogues should be the same as the wild-type version of MENTSH, we cannot exclude the possibility that these amino acid changes have led to a gain of function mutation that activates novel pathways.

A previous report has identified different sub-types of T2D in Mexican cohorts 54 and another has found that different sub-types of T2D may have an underlying genetic component 55. Future studies may find that the MENTSH SNP is an underlying cause of specific T2D sub-types and thus would lead to a more precision medicine approach to treating T2D in carriers.

## Conclusions

Our observations have several important implications for patients with diabetes and obesity. First, MENTSH represents a novel regulator of metabolism that may have translational implications. The differential and opposite effects of MENTSH and its analogues on fat versus muscle are unique and may lead to novel therapeutic avenues. Furthermore, MENTSH represents a potential opportunity to predict the risk of developing diabetes in susceptible individuals who can easily determine their SNP status and employ lifestyle preventive measures. It remains to be determined if the MENTSH SNP determines the response to specific therapies that are currently available.

## Supplementary Material

Supplementary figures and tables.

## Data Availability

All data is available upon request to the corresponding author.

## Abbreviations

BMI: body mass index
CHD: coronary heart disease
DIO: diet induced obesity
EIF2: elongation initiation factor 2
Gee1: general estimating equations
GTT: glucose tolerance test
HbA1c: hemoglobin A1c
HRS: Health and Retirement Study
HPLC: high-performance liquid chromatography
HTN-IR: Hypertension-Insulin Resistance Family Study
IP: intra-peritoneal
ITT: insulin tolerance test
LR: linear regression
MSD: Meso Scale Discovery
MDP: mitochondrial derived microprotein
MENTSH: MDP encoded in the ND-two subunit of humans
MESA: Multi-Ethnic Study of Atherosclerosis Cohort
MiWIS: mitochondrial DNA wide interaction study
mtDNA: mitochondrial DNA
mtPCs: mitochondrial principal components
NHW: non-hispanic whites
nucPCs: nuclear principal components
OCR: oxygen consumption rate
PCA: principal component analysis
RER: respiratory exchange rate
S.E.M.: standard error of the mean
SNP: single nucleotide polymorphism
T2D: type 2 diabetes

## References

[1] Stokes A, Preston SH (2017). Deaths Attribux to Diabetes in the United States: Comparison of Data Sources and Estimation Approaches. PLoS One.

[2] Herrington WG, Alegre-Diaz J, Wade R, Gnatiuc L, Ramirez-Reyes R, Hill M (2018). Effect of diabetes duration and glycaemic control on 14-year cause-specific mortality in Mexican adults: a blood-based prospective cohort study. Lancet Diabetes Endocrinol.

[3] Arredondo A (2018). Diabetes duration, HbA1c, and cause-specific mortality in Mexico. Lancet Diabetes Endocrinol.

[4] Wang MC, Shah NS, Carnethon MR, O'Brien MJ, Khan SS (2021). Age at Diagnosis of Diabetes by Race and Ethnicity in the United States From 2011 to 2018. JAMA Intern Med.

[5] Collaborators GUHD (2023). Cause-specific mortality by county, race, and ethnicity in the USA, 2000-19: a systematic analysis of health disparities. Lancet.

[6] Bielinski SJ, Yanes Cardozo LL, Takahashi PY, Larson NB, Castillo A, Podwika A (2023). Predictors of Metformin Failure: Repurposing Electronic Health Record Data to Identify High-Risk Patients. J Clin Endocrinol Metab.

[7] Consortium STD, Williams AL, Jacobs SB, Moreno-Macias H, Huerta-Chagoya A, Churchhouse C (2014). Sequence variants in SLC16A11 are a common risk factor for type 2 diabetes in Mexico. Nature.

[8] Lara-Riegos JC, Ortiz-López MG, Peña-Espinoza BI, Montúfar-Robles I, Peña-Rico MA, Sánchez-Pozos K (2015). Diabetes susceptibility in Mayas: Evidence for the involvement of polymorphisms in HHEX, HNF4α, KCNJ11, PPARγ, CDKN2A/2B, SLC30A8, CDC123/CAMK1D, TCF7L2, ABCA1 and SLC16A11 genes. Gene.

[9] Traurig M, Hanson RL, Marinelarena A, Kobes S, Piaggi P, Cole S (2016). Analysis of SLC16A11 Variants in 12,811 American Indians: Genotype-Obesity Interaction for Type 2 Diabetes and an Association with RNASEK Expression. Diabetes.

[10] Bowden DW, An SS, Palmer ND, Brown WM, Norris JM, Haffner SM (2010). Molecular basis of a linkage peak: exome sequencing and family-based analysis identify a rare genetic variant in the ADIPOQ gene in the IRAS Family Study. Hum Mol Genet.

[11] Qi Q, Stilp AM, Sofer T, Moon JY, Hidalgo B, Szpiro AA (2017). Genetics of Type 2 Diabetes in U.S. Hispanic/Latino Individuals: Results from the Hispanic Community Health Study/Study of Latinos (HCHS/SOL). Diabetes.

[12] Zhang S, Reljic B, Liang C, Kerouanton B, Francisco JC, Peh JH (2020). Mitochondrial peptide BRAWNIN is essential for vertebrate respiratory complex III assembly. Nat Commun.

[13] Chu Q, Martinez TF, Novak SW, Donaldson CJ, Tan D, Vaughan JM (2019). Regulation of the ER stress response by a mitochondrial microprotein. Nat Commun.

[14] Kondo T, Hashimoto Y, Kato K, Inagaki S, Hayashi S, Kageyama Y (2007). Small peptide regulators of actin-based cell morphogenesis encoded by a polycistronic mRNA. Nat Cell Biol.

[15] Kondo T, Plaza S, Zanet J, Benrabah E, Valenti P, Hashimoto Y (2010). Small peptides switch the transcriptional activity of Shavenbaby during Drosophila embryogenesis. Science.

[16] Kienzle L, Bettinazzi S, Choquette T, Brunet M, Khorami HH, Jacques JF (2023). A small protein coded within the mitochondrial canonical gene nd4 regulates mitochondrial bioenergetics. BMC Biol.

[17] Martinez TF, Lyons-Abbott S, Bookout AL, De Souza EV, Donaldson C, Vaughan JM (2023). Profiling mouse brown and white adipocytes to identify metabolically relevant small ORFs and functional microproteins. Cell Metab.

[18] Chen Y, Cao X, Loh KH, Slavoff SA (2023). Chemical labeling and proteomics for characterization of unannotated small and alternative open reading frame-encoded polypeptides. Biochem Soc Trans.

[19] Makarewich CA, Munir AZ, Bezprozvannaya S, Gibson AM, Young Kim S, Martin-Sandoval MS (2022). The cardiac-enriched microprotein mitolamban regulates mitochondrial respiratory complex assembly and function in mice. Proc Natl Acad Sci U S A.

[20] Cao X, Khitun A, Harold CM, Bryant CJ, Zheng SJ, Baserga SJ (2022). Nascent alt-protein chemoproteomics reveals a pre-60S assembly checkpoint inhibitor. Nat Chem Biol.

[21] Kim SJ, Miller B, Hartel NG, Ramirez R 2nd, Braniff RG, Leelaprachakul N (2024). A naturally occurring variant of SHLP2 is a protective factor in Parkinson's disease. Mol Psychiatry.

[22] Jia H, Zhou LC, Chen YF, Zhang W, Qi W, Wang P (2024). Mitochondria-encoded peptide MOTS-c participates in plasma membrane repair by facilitating the translocation of TRIM72 to membrane. Theranostics.

[23] Zou R, Shi W, Chang X, Zhang M, Tan S, Li R (2024). The DNA-dependent protein kinase catalytic subunit exacerbates endotoxemia-induced myocardial microvascular injury by disrupting the MOTS-c/JNK pathway and inducing profilin-mediated lamellipodia degradation. Theranostics.

[24] Hashimoto Y, Niikura T, Tajima H, Yasukawa T, Sudo H, Ito Y (2001). A rescue factor abolishing neuronal cell death by a wide spectrum of familial Alzheimer's disease genes and Abeta. Proc Natl Acad Sci U S A.

[25] Lee C, Zeng J, Drew BG, Sallam T, Martin-Montalvo A, Wan J (2015). The mitochondrial-derived peptide MOTS-c promotes metabolic homeostasis and reduces obesity and insulin resistance. Cell Metab.

[26] Cobb LJ, Lee C, Xiao J, Yen K, Wong RG, Nakamura HK (2016). Naturally occurring mitochondrial-derived peptides are age-dependent regulators of apoptosis, insulin sensitivity, and inflammatory markers. Aging (Albany NY).

[27] Miller B, Kim SJ, Mehta HH, Cao K, Kumagai H, Thumaty N (2023). Mitochondrial DNA variation in Alzheimer's disease reveals a unique microprotein called SHMOOSE. Mol Psychiatry.

[28] Kim KH (2023). Intranasal delivery of mitochondrial protein humanin rescues cell death and promotes mitochondrial function in Parkinson's disease. Theranostics.

[29] Weir DR (2013). HRS: Quality control report for genotypic data. St. Louis, MO: University of Washington.

[32] Miller B, Arpawong TE, Jiao H, Kim SJ, Yen K, Mehta HH (2019). Comparing the Utility of Mitochondrial and Nuclear DNA to Adjust for Genetic Ancestry in Association Studies. Cells.

[33] Chang CC, Chow CC, Tellier LC, Vattikuti S, Purcell SM, Lee JJ (2015). Second-generation PLINK: rising to the challenge of larger and richer datasets. Gigascience.

[34] Bild DE, Bluemke DA, Burke GL, Detrano R, Diez Roux AV, Folsom AR (2002). Multi-Ethnic Study of Atherosclerosis: objectives and design. Am J Epidemiol.

[35] Xiang AH, Azen SP, Raffel LJ, Tan S, Cheng LS, Diaz J (2001). Evidence for joint genetic control of insulin sensitivity and systolic blood pressure in hispanic families with a hypertensive proband. Circulation.

[36] Gunderson KL, Steemers FJ, Ren H, Ng P, Zhou L, Tsan C (2006). Whole-genome genotyping. Methods Enzymol.

[37] Shen R, Fan JB, Campbell D, Chang W, Chen J, Doucet D (2005). High-throughput SNP genotyping on universal bead arrays. Mutat Res.

[38] Palmer ND, Goodarzi MO, Langefeld CD, Wang N, Guo X, Taylor KD (2015). Genetic Variants Associated with Quantitative Glucose Homeostasis Traits Translate to Type 2 Diabetes in Mexican Americans: The GUARDIAN (Genetics Underlying Diabetes in Hispanics) Consortium. Diabetes.

[39] Berridge MV, Tan AS (1993). Characterization of the cellular reduction of 3-(4,5-dimethylthiazol-2-yl)-2,5-diphenyltetrazolium bromide (MTT): subcellular localization, substrate dependence, and involvement of mitochondrial electron transport in MTT reduction. Arch Biochem Biophys.

[40] Brandhorst S, Choi IY, Wei M, Cheng CW, Sedrakyan S, Navarrete G (2015). A Periodic Diet that Mimics Fasting Promotes Multi-System Regeneration, Enhanced Cognitive Performance, and Healthspan. Cell Metab.

[41] Kim SJ, Miller B, Mehta HH, Xiao J, Wan J, Arpawong TE (2019). The mitochondrial-derived peptide MOTS-c is a regulator of plasma metabolites and enhances insulin sensitivity. Physiol Rep.

[42] Juster FT, Suzman R (1995). An Overview of the Health and Retirement Study. J Hum Resour.

[43] Salas A, Lovo-Gomez J, Alvarez-Iglesias V, Cerezo M, Lareu MV, Macaulay V (2009). Mitochondrial echoes of first settlement and genetic continuity in El Salvador. PLoS One.

[44] Jumper J, Evans R, Pritzel A, Green T, Figurnov M, Ronneberger O (2021). Highly accurate protein structure prediction with AlphaFold. Nature.

[45] Chin YP, Keni J, Wan J, Mehta H, Anene F, Jia Y (2013). Pharmacokinetics and tissue distribution of humanin and its analogues in male rodents. Endocrinology.

[46] Cundy KC, Grinstaff KK, Magnan R, Luo W, Yao Y, Yan LZ (2023). Therapeutic Peptides. In: USPTO, editor. United States.

[47] Lee C, Wan J, Miyazaki B, Fang Y, Guevara-Aguirre J, Yen K (2014). IGF-I regulates the age-dependent signaling peptide humanin. Aging Cell.

[48] Zhao Y, Feng Z, Zhang Y, Sun Y, Chen Y, Liu X (2019). Gain-of-Function Mutations of SLC16A11 Contribute to the Pathogenesis of Type 2 Diabetes. Cell Rep.

[49] Rusu V, Hoch E, Mercader JM, Tenen DE, Gymrek M, Hartigan CR (2017). Type 2 Diabetes Variants Disrupt Function of SLC16A11 through Two Distinct Mechanisms. Cell.

[50] Cao X, Khitun A, Na Z, Dumitrescu DG, Kubica M, Olatunji E (2020). Comparative Proteomic Profiling of Unannotated Microproteins and Alternative Proteins in Human Cell Lines. J Proteome Res.

[51] Hevener AL, Zhou Z, Moore TM, Drew BG, Ribas V (2018). The impact of ERalpha action on muscle metabolism and insulin sensitivity - Strong enough for a man, made for a woman. Mol Metab.

[52] Shi Y, Taylor SI, Tan SL, Sonenberg N (2003). When translation meets metabolism: multiple links to diabetes. Endocr Rev.

[53] Ko SH, Cho B, Shin D (2025). Microproteins in Metabolic Biology: Emerging Functions and Potential Roles as Nutrient-Linked Biomarkers. Int J Mol Sci.

[54] Bello-Chavolla OY, Bahena-Lopez JP, Vargas-Vazquez A, Antonio-Villa NE, Marquez-Salinas A, Fermin-Martinez CA (2020). Clinical characterization of data-driven diabetes subgroups in Mexicans using a reproducible machine learning approach. BMJ Open Diabetes Res Care.

[55] Mansour Aly D, Dwivedi OP, Prasad RB, Karajamaki A, Hjort R, Thangam M (2021). Genome-wide association analyses highlight etiological differences underlying newly defined subtypes of diabetes. Nat Genet.

