# Supplementary material for: MENTSH: A novel mitochondrial microprotein linked to a SNP associated with type 2 diabetes
Source: Theranostics. 2026 Jul 20;16(14):8180–94. doi: 10.7150/thno.134637 (PMC13440476; doi:10.7150/thno.134637)
Supplement: Supplementary file 1 [file thnov16p8180s1.pdf]

Supplementary Materials

Supplemental Figure 1

A.

```
att aaa cca aac cca gct acg caa aat ctt
 M   K   P   N   P   A   T   Q   N   L
agc ata ctc ctc aat tac cca cat agg
 S   M   L   L   N   Y   P   H   -
```

B.

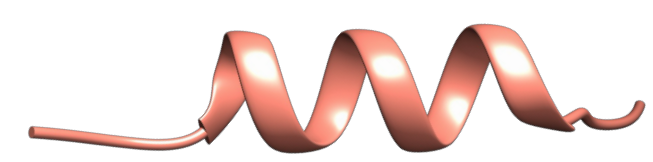

Figure S1. (**A**) The nucleotide sequence and mitochondrial translation of MENTSH. The location of the human SNP is highlighted in red. (**B**) The predicted 3D structure of MENTSH as predicted by AlphaFold.

Supplemental Figure 2

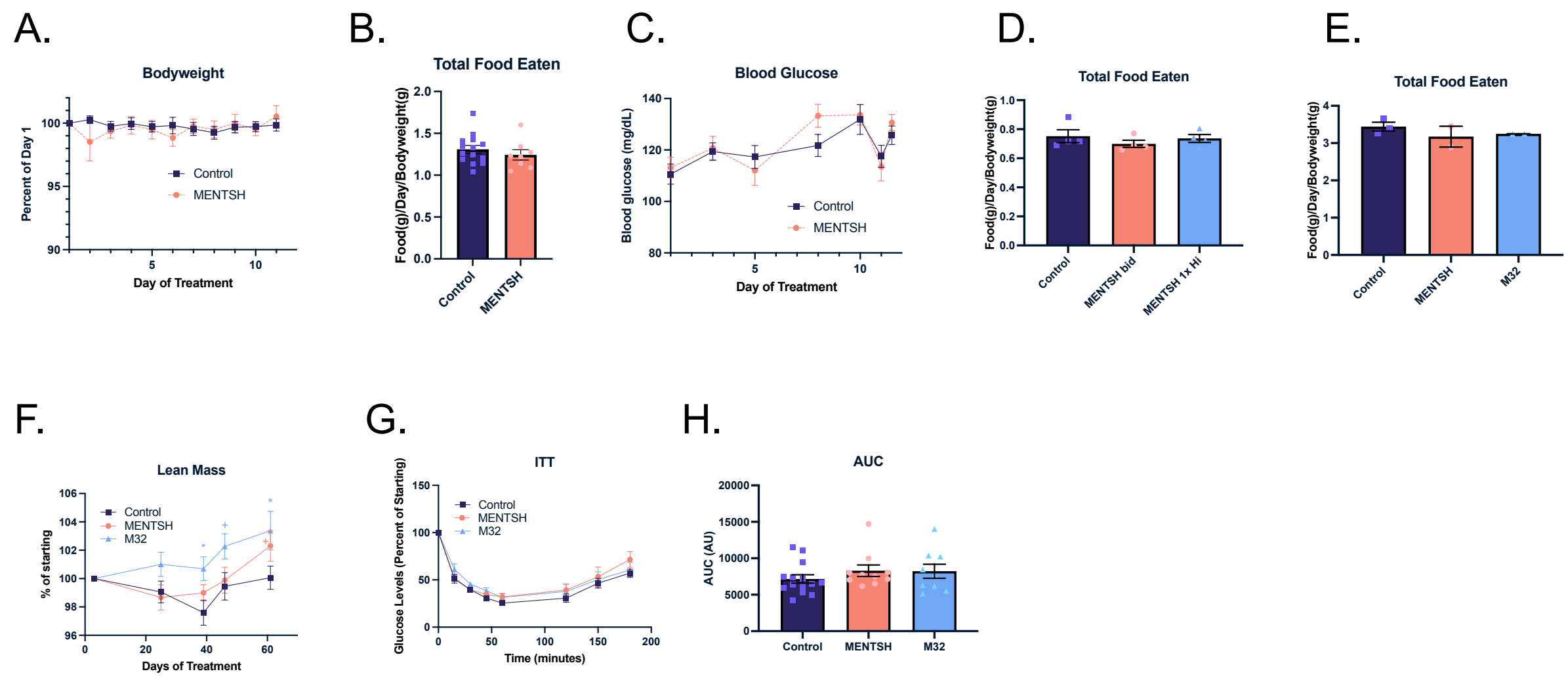

Figure S2. Similar to the data in humans, MENTSH only has an effect in mice on a high fat diet and on a normal, chow diet, it has no effect on (**A**) body weight, (**B**) food consumption, (**C**) nor blood glucose (n = 14 for control, N = 8 for MENTSH treated). Mice on a high fat diet for 10 days treated with either MENTSH (2.5 mg/kg bid) or MENTSH 1x/day (20mg/kg) did not have (**D**) a change in total food consumed (N = 4 cages/group). In a long-term study, although bodyweight gain was significantly attenuated Figure 2E, (**E**) food consumption was not significantly different (N = 3,2,2/cage for Control, MENTSH, and M32 respectively). In the same study, (**F**) lean mass was significantly different from control in the M32 group and trended towards a difference by the end of the study in the MENTSH treated group. An insulin tolerance test (ITT) were performed on these animals on day 50 of treatment (**G**, **H**) with no significant difference between all groups. Error bars represent s.e.m. * $p < .05$, +$p < .1$

Supplement for Figure 3

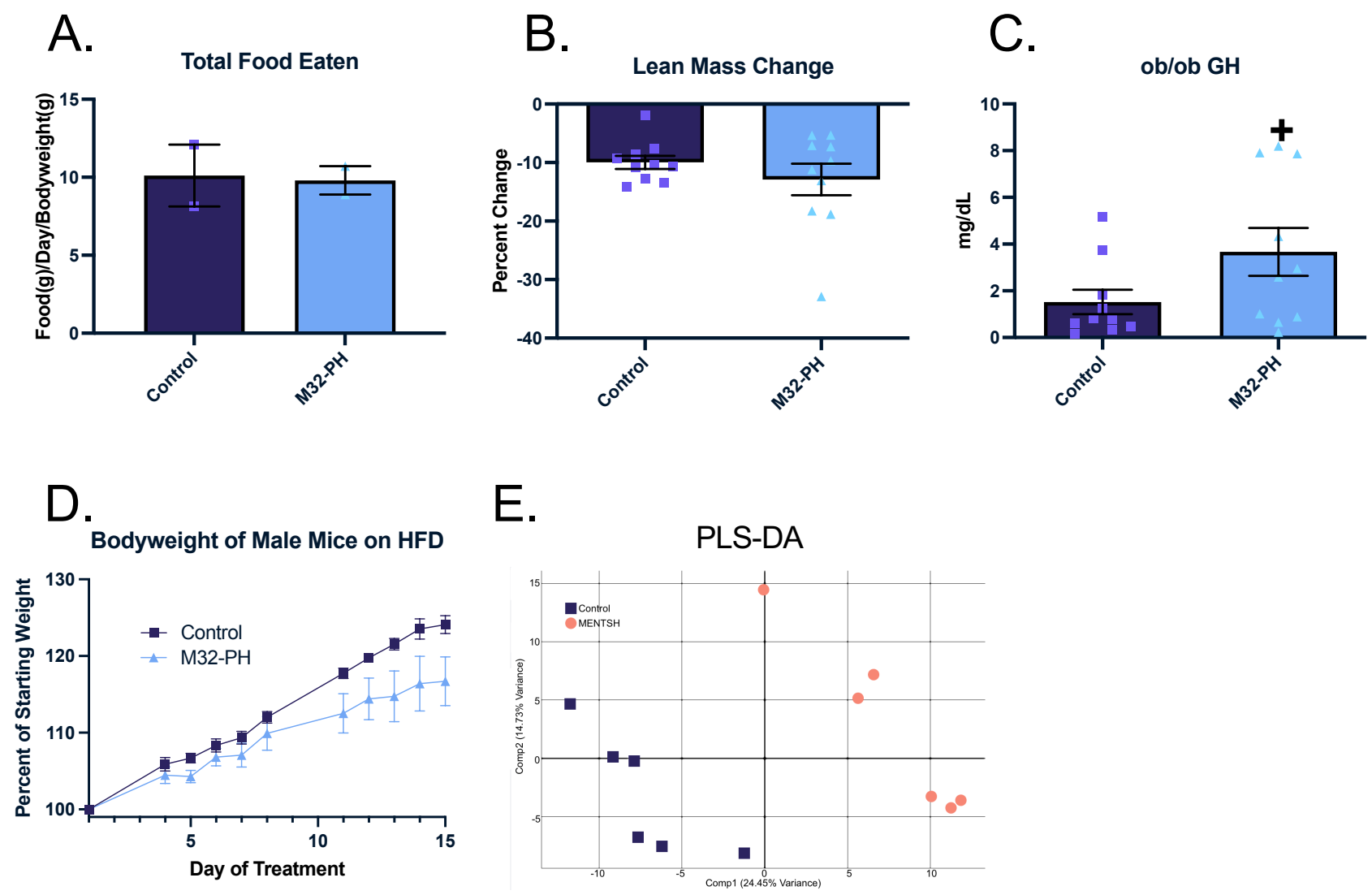

Figure S3.
(**A**) Treatment of ob/ob mice with M32-PH did not change food intake in mice. (**B**) Treatment also did not cause a significant change in lean body mass. (**C**) Circulating GH trended towards an increase (p = .0911). (**D**) Alzet pumps filled with either PBS or M32-PH (2.5 mg/kg/day) were implanted subcutaneously to (**D**) male mice on a HFD (N = 5/group). The slope of each line is significantly different from each other (p = .0416). (**F**) Partial least squares discriminant analysis of metabolomic data. Error bars represent s.e.m.

Supplementary Figure 4

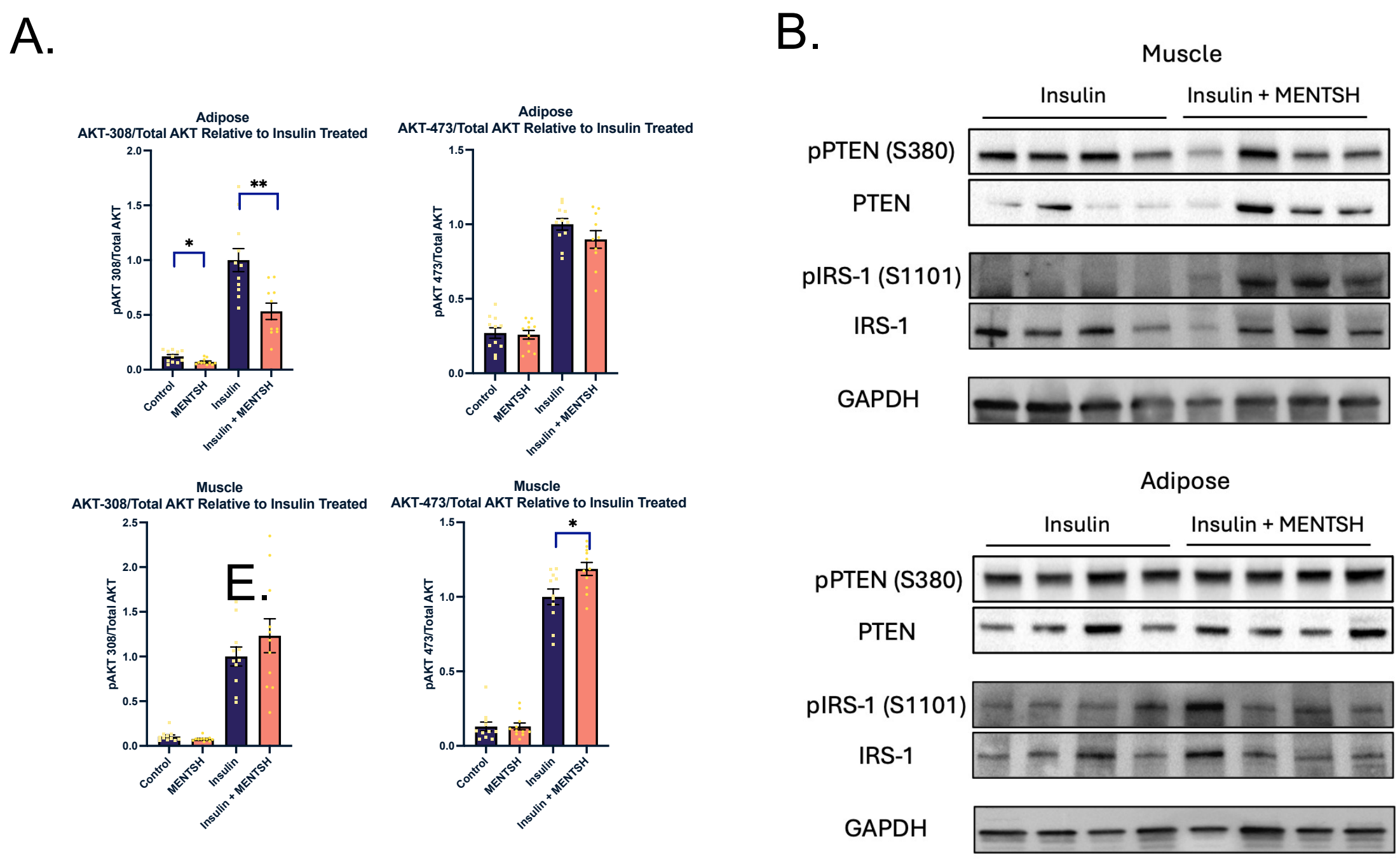

Figure S4. (**A**) Phospho-AKT results from Figure 4A and 4B. Data is relative to insulin treated control. (**B**) Western Blots from Figure 4C and 4D. Error bars represent s.e.m. * indicates p < .05

Supplementary Figure 5

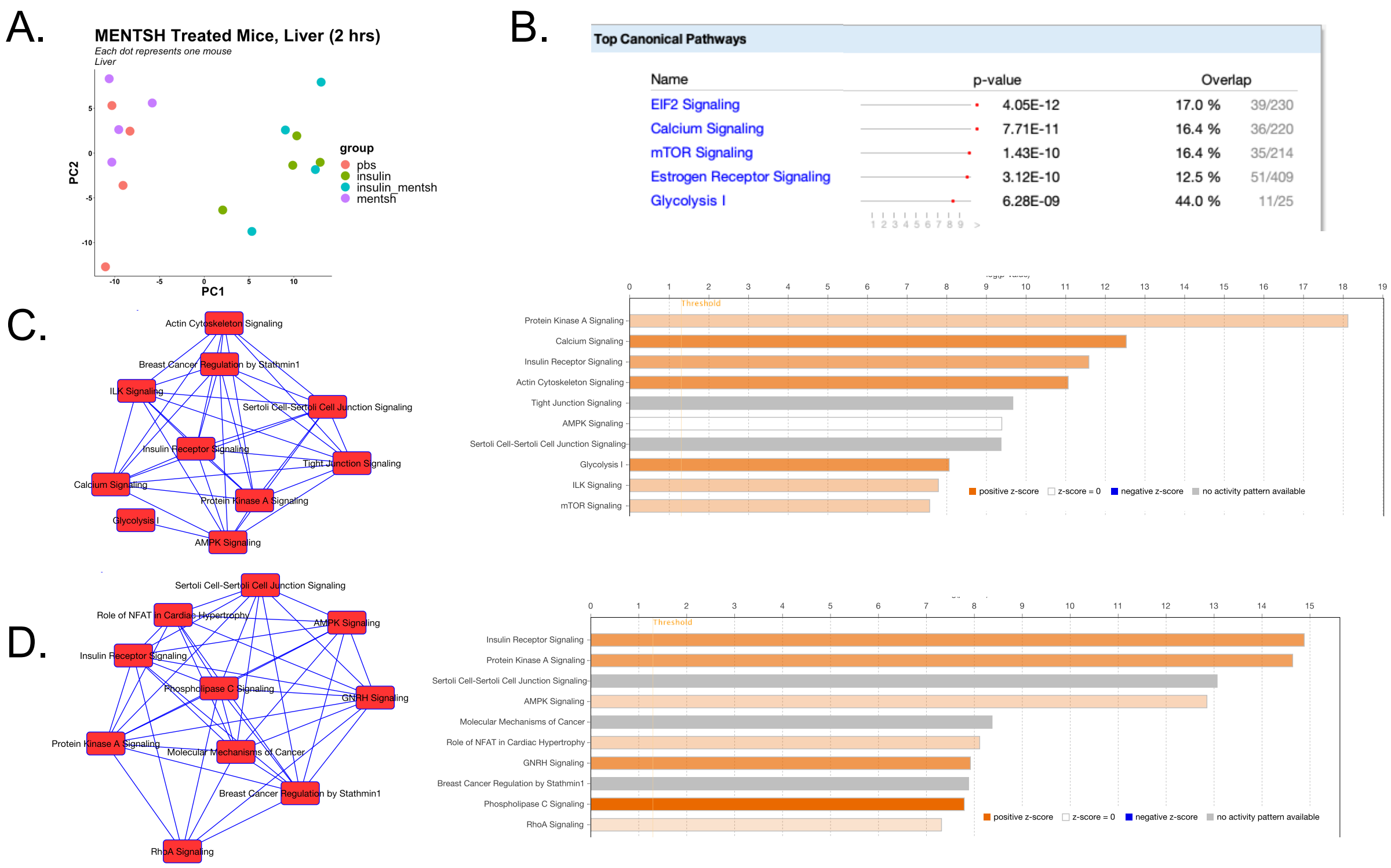

Figure S5. (**A**) A PCA plot of RNAseq data of the livers of mice treated with MENTSH could not discriminate MENTSH and Control treated groups. (**B**) IPA pathway analysis of the insulin +/- MENTSH muscle samples indicated that some of the top pathways were metabolism related. (**C**) Pathway analysis of muscle tissues treated with MENTSH. (**D**) Pathway analysis of adipose tissues treated with MENTSH.

Table S1. List of all the MENTSH analogues that were screened or used in these experiments.

	Sequence
MENTSH	MKPNPATQNLSMLLNYPH
1	AKPNPATQNLSMLLNYPH
2	MAPNPATQNLSMLLNYPH
3	MKANPATQNLSMLLNYPH
4	MKPAPATQNLSMLLNYPH
5	MKPNAATQNLSMLLNYPH
6	MKPNPATQNLSMLLNYPH
7	MKPNPAAQNLSMLLNYPH
8	MKPNPATANLSMLLNYPH
9	MKPNPATQALSMLLNYPH
10	MKPNPATQNASMLLNYPH
11	MKPNPATQNLAMLLNYPH
12	MKPNPATQNLSALLNYPH
13	MKPNPATQNLSMALNYPH
14	MKPNPATQNLSMLANYPH
15	MKPNPATQNLSMLLAYPH
16	MKPNPATQNLSMLLNAPH
17	MKPNPATQNLSMLLNYAH
18	MKPNPATQNLSMLLNYPA
19	KPNPATQNLSMLLNYPH
20	(Nle)KPNPATQNLS(Nle)LLNYPH
21	MKPNPATQN
22	PATQNLSML
23	NLSMLLNYP
24	LLNYPH
25	MKANAATQNLSMLLNYAH
26	MKGNGATQNLSMLLNYGH

27	MKANAATQNLSMLLNYPH
28	MKPNAATQNLSMLLNYAH
29	MKANPATQNLSMLLNYAH
30	MKPNPADQNLDMLLNDPH
31	MKPNPADQNLDMLLNYPH
32/M32	MKPNPATQNLDMLLNDPH
33	MKPNPADQNLSMLLNDPH
34	MKPNPADQNLSMLLNYPH
35	MKPNPATQNLDMLLNYPH
36	MKPNPATQNLSMLLNDPH
37	MKPNPAGQNLGMLLNGPH
38	MKPNPAGQNLGMLLNYPH
39	MKPNPATQNLGMLLNGPH
40	MKPNPAGQNLSMLLNGPH
41	MKPNPAGQNLSMLLNYPH
42	MKPNPATQNLGMLLNYPH
43	MKPNPATQNLSMLLNGPH
44	MKPNPSTQNTSMTTNQPH
45	MKPNPSTQNTSMTTNYPH
46	MKPNPSTQNTSMTLNQPH
47	MKPNPSTQNTSMLTNQPH
48	MKPNPATQNTSMTTNQPH
49	MKPNPSTQNTSMTLNYPH
50	MKPNPATQNTSMTTNYPH
51	MKPNPATQNLSMTTNQPH
52	MKPNPSTQNLSMLTNQPH
53	MKPNPSTQNTSMLSNQPH
54	MKPNPATQNTSMLTNQPH
55	MKPNPSTQNLSMTLNQPH
56	MKPNPSTQNLSMTTNYPH

57	MKPNPATQNTSMTLNQPH
58	MKPNPSTQNTSMLTNYPH
59	MKPNPSTQNTSMLLNYPH
60	MKPNPSTQNLSMTLNYPH
61	MKPNPSTQNLSMLTNYPH
62	MKPNPSTQNLSMLLNQPH
63	MKPNPLTQNTSMTLNYPH
64	MKPNPLTQNTSMLTNYPH
65	MKPNPATQNTSMLLNQPH
66	MKPNPLTQNLSMTTNYPH
67	MKPNPLTQNLSMTLNQPH
68	MKPNPATQNLSMLTNQPH
69	MKPNPSTQNLSMLLNYPH
70	MKPNPATQNTSMLLNYPH
71	MKPNPATQNLSMTLNYPH
72	MKPNPATQNLSMLTNYPH
73	MKPNPATQNLSMLLNQPH
74	AKPNPATANLSMLLNYPA
75	MAPNPATQNASMLLNYAH
76	MKPAPATANLSMLLNYAH
77	MKPAPATQALSMLLAYPH
78	MKPNAATQALSMLANYPH
79	MAPNPAAQNLSALLNYPH
80	MKPNPATQNLSMALAYAH
81	AKPAPAAQNLSMLLNYPH
82	MKPNPATQNLSMALAYPA
83	MKPNAATANLAMLLNYPH
84	MKPNPATQALSMALNAPH
85	MAPNAATQNASMLLNYPH
86	AKANPATQNLSMLLNYPH

87	MAPAPATQNLSMLLNYPH
88	MKPAPAAQNLSMLLNYPH
89	MKPNAAAQNLSMLLNYPH
90	MKPNPATANASMLLNYPH
91	MKPNPATQALAMLLNYPH
92	MKPNPATQNASALLNYPH
93	MKPNPATQNLAMALNYPH
94	MKPNPATQNLSALANYPH
95	MKPNPATQNLSMALAYPH
96	MKPNPATQNLSMLANAPH
M32-PH	MKPNPATQNLDMLLND

Table S2. List of all the lipid related metabolites found in the metabolomic experiment

Sub Pathway	Biochemical Name	PUBCHEM	MENTSH Ctrl	p-value	q-value
Medium Chain Fatty Acid	caproate (6:0)	8892	1.33	0.1322	0.2787
	heptanoate (7:0)	8094	1.36	0.1255	0.2787
	caprylate (8:0)	379	1.72	0.0466	0.1915
	10-undecenoate (11:1n1)		0.98	0.9279	0.5602
	5-dodecenoate (12:1n7)	5312378	0.77	0.1227	0.276
Polyunsaturated Fatty Acid (n3 and n6)	stearidonate (18:4n3)	5312508	0.82	0.1966	0.3287
	eicosapentaenoate (EPA; 20:5n3)	446284	0.57	0.0098	0.0876
	docosapentaenoate (n3 DPA; 22:5n3)	6441454	0.71	0.0771	0.2342
	docosahexaenoate (DHA; 22:6n3)	445580	0.67	0.0216	0.123
	linoleate (18:2n6)	5280450	0.89	0.2686	0.3573
	linolenate [alpha or gamma; (18:3n3 or 6)]	5280934	0.76	0.0821	0.238
	dihomo-linolenate (20:3n3 or n6)	5280581	0.73	0.1168	0.2679
	arachidonate (20:4n6)	444899	0.71	0.0927	0.251
	adrenate (22:4n6)	5497181	0.64	0.1222	0.276
	docosapentaenoate (n6 DPA; 22:5n6)	6441454	0.59	0.0915	0.251
	docosadienoate (22:2n6)	5282807	0.94	0.8981	0.5506
	dihomo-linoleate (20:2n6)	6439848	0.76	0.1085	0.26
	mead acid (20:3n9)	5312531	0.64	0.1908	0.3266
Fatty Acid, Amino	2-aminoheptanoate	227939	0.33	0.02	0.1203
	2-aminooctanoate	69522	1.07	0.5225	0.458
Fatty Acid Synthesis	malonate	867	0.78	0.0158	0.106
Fatty Acid Metabolism (also BCAA Metabolism)	butyrylcarnitine	439829	0.79	0.2757	0.3602
	butyrylglycine	88412	0.87	0.3001	0.3675
	propionylcarnitine	107738	1.08	0.4602	0.4246
	propionylglycine	98681	1.64	0.008	0.0867
	methylmalonate (MMA)	487	1.11	0.7253	0.5138
Fatty Acid Metabolism(Acyl Carnitine)	acetylcarnitine	1	0.87	0.3602	0.3892
	3-hydroxybutyrylcarnitine (1)	53481617	0.52	0.0281	0.147
	3-hydroxybutyrylcarnitine (2)		0.87	0.5559	0.4675
	hexanoylcarnitine	6426853	0.7	0.1995	0.3313
	octanoylcarnitine	123701	0.79	0.3866	0.393
	decanoylcarnitine	10245190	0.73	0.1025	0.2553

	cis-4-decenoyl carnitine		0.92	0.877	0.5449
	laurylcarnitine	10427569	**0.69**	0.0505	0.1987
	myristoylcarnitine	53477791	**0.67**	0.0114	0.0935
	palmitoylcarnitine	461	**0.78**	0.0165	0.1075
	palmitoleoylcarnitine*		**0.6**	0.0018	0.0441
	stearoylcarnitine	6426855	0.91	0.2834	0.3621
	linoleoylcarnitine*	6450015	**0.69**	0.0039	0.0655
	oleoylcarnitine	6441392;53477789	**0.73**	0.0286	0.1471
	myristoleoylcarnitine*		**0.68**	0.0156	0.106
	adipoylcarnitine		**0.52**	0.0004	0.014
	pimeloylcarnitine/3-methyladipoylcarnitine		1.95	0.3649	0.3904
Fatty Acid, Monohydroxy	2-hydroxyoctanoate	94180	1.17	0.5668	0.4729
	3-hydroxyhexanoate	151492	0.7	0.228	0.3477
	3-hydroxyoctanoate	26613	0.89	0.638	0.4902
	3-hydroxydecanoate	26612	0.89	0.5401	0.4616
	3-hydroxysebacate	3017884	0.86	0.9821	0.5768
	3-hydroxylaurate	94216	0.74	0.1402	0.2804
	3-hydroxymyristate	16064	**0.81**	0.0963	0.2515
	5-hydroxyhexanoate	170748	**1.98**	0.0453	0.1898
	8-hydroxyoctanoate	69820	1.18	0.2673	0.3573
	16-hydroxypalmitate	10466	1.2	0.3129	0.3726
	13-HODE + 9-HODE	43013	0.71	0.162	0.3055
	14-HDoHE/17-HDoHE		**0.65**	0.075	0.2342
Lysolipid	1-palmitoyl-GPC (16:0)	86554	0.95	0.423	0.4105
	2-palmitoyl-GPC (16:0)*	15061532	**0.8**	0.0756	0.2342
	1-palmitoleoyl-GPC (16:1)*	24779461	**0.79**	0.014	0.1044
	1-stearoyl-GPC (18:0)	497299	**0.85**	0.0477	0.1924
	1-oleoyl-GPC (18:1)	16081932	**0.89**	0.0645	0.218
	1-linoleoyl-GPC (18:2)	11988421	0.89	0.1967	0.3287
	1-linolenoyl-GPC (18:3)*		0.86	0.2999	0.3675
	1-arachidonoyl-GPC (20:4n6)*		0.7	0.1773	0.3169
	1-lignoceroyl-GPC (24:0)		0.79	0.318	0.3727
	1-palmitoyl-GPE (16:0)	9547069	**0.8**	0.0932	0.251
	1-stearoyl-GPE (18:0)	9547068	**0.74**	0.0049	0.0736
	2-stearoyl-GPE (18:0)*		**0.52**	0.0181	0.1153
	1-oleoyl-GPE (18:1)	9547071	**0.84**	0.0545	0.2045
	1-linoleoyl-GPE (18:2)*	52925130	0.9	0.3872	0.393
	1-arachidonoyl-GPE (20:4n6)*	42607465	**0.71**	0.0245	0.1324
	1-stearoyl-GPI (18:0)		**0.64**	0.0361	0.1697

	1-arachidonoyl-GPI (20:4)*		0.62	0.1107	0.2634
	1-linoleoyl-GPG (18:2)*		0.87	0.4453	0.4175
	1-linoleoyl-GPA (18:2)*		**0.73**	0.0194	0.1189
Lysoplasmalogen	1-(1-enyl-palmitoyl)-GPC (P-16:0)*	10917802	**0.82**	0.0085	0.0867
	1-(1-enyl-palmitoyl)-GPE (P-16:0)*		**0.76**	0.0101	0.0876
	1-(1-enyl-oleoyl)-GPE (P-18:1)*		**0.74**	0.0537	0.2039
	1-(1-enyl-stearoyl)-GPE (P-18:0)*		**0.75**	0.0114	0.0935
Monoacylglycerol	1-palmitoylglycerol (16:0)	14900	0.9	0.7961	0.5302
	2-palmitoylglycerol (16:0)	123409	1.36	0.3339	0.3785
	1-oleoylglycerol (18:1)	5283468	**0.66**	0.0391	0.1709
	2-oleoylglycerol (18:1)	5319879	**0.74**	0.0638	0.218
	1-linoleoylglycerol (18:2)	5283469	**0.76**	0.0081	0.0867
	2-linoleoylglycerol (18:2)	5365676	**0.8**	0.0655	0.218
	1-linolenoylglycerol (18:3)	53480978	**0.63**	0.0379	0.1697
	1-arachidonylglycerol (20:4)	5282281	0.87	0.2671	0.3573
	1-docosahexaenoylglycerol (22:6)		**0.38**	0.002	0.0459
	1-dihomo-linolenylglycerol (20:3)		0.82	0.3699	0.392
	1-palmitoleoylglycerol (16:1)*		0.75	0.2365	0.3569
Diacylglycerol	diacylglycerol (14:0/18:1, 16:0/16:1) [1]*		**0.54**	0.0187	0.1171
	diacylglycerol (14:0/18:1, 16:0/16:1) [2]*		0.7	0.1483	0.2901
	diacylglycerol (16:1/18:2 [2], 16:0/18:3 [1])*		**0.71**	0.0552	0.2045
	oleoyl-linoleoyl-glycerol (18:1/18:2) [1]		0.94	0.8087	0.5337
	oleoyl-linoleoyl-glycerol (18:1/18:2) [2]		0.97	0.7144	0.5138
	oleoyl-linolenoyl-glycerol (18:1/18:3) [2]*		**0.73**	0.0631	0.218
	oleoyl-arachidonoyl-glycerol (18:1/20:4) [2]*		**0.7**	0.0155	0.106
	linoleoyl-arachidonoyl-glycerol (18:2/20:4) [1]*		**0.69**	0.0235	0.1289
	linoleoyl-arachidonoyl-glycerol (18:2/20:4) [2]*		**0.72**	0.0128	0.1016
	linoleoyl-linoleoyl-glycerol (18:2/18:2) [1]*		1.04	0.6597	0.4995
	linoleoyl-linolenoyl-glycerol (18:2/18:3) [1]*		0.8	0.216	0.3408
	linoleoyl-linolenoyl-glycerol (18:2/18:3) [2]*		0.87	0.5314	0.459
	linoleoyl-docosahexaenoyl-glycerol (18:2/22:6) [1]*		**0.65**	0.0213	0.123
	linoleoyl-docosahexaenoyl-glycerol (18:2/22:6) [2]*		**0.72**	0.009	0.0867
	palmitoyl-arachidonoyl-glycerol (16:0/20:4) [1]*		**0.42**	0.0994	0.2524
	palmitoyl-arachidonoyl-glycerol (16:0/20:4) [2]*		0.66	0.1325	0.2787
	palmitoleoyl-linoleoyl-glycerol (16:1/18:2) [1]*		**0.62**	0.0139	0.1044
	palmitoyl-oleoyl-glycerol (16:0/18:1) [1]*		**0.81**	0.0972	0.252
	palmitoyl-oleoyl-glycerol (16:0/18:1) [2]*		0.86	0.2423	0.3569
	palmitoyl-palmitoyl-glycerol (16:0/16:0) [2]*		0.91	0.6417	0.4916

	palmitoyl-linoleoyl-glycerol (16:0/18:2) [1]*	9543695	0.87	0.2348	0.3565
	palmitoyl-linoleoyl-glycerol (16:0/18:2) [2]*		0.88	0.2863	0.3621
	palmitoyl-linolenoyl-glycerol (16:0/18:3) [2]*		0.58	0.1337	0.2787
	stearoyl-linoleoyl-glycerol (18:0/18:2) [2]*		1.14	0.3992	0.3989
	oleoyl-oleoyl-glycerol (18:1/18:1) [1]*		0.94	0.9215	0.5598
	oleoyl-oleoyl-glycerol (18:1/18:1) [2]*		0.93	0.8445	0.5416
Sphingolipid Metabolism	sphinganine	3126	**0.59**	0.0141	0.1044
	sphinganine-1-phosphate	520	**0.73**	0.0225	0.1258
	N-palmitoyl-sphinganine (d18:0/16:0)	5283572	0.82	0.249	0.3573
	palmitoyl dihydrosphingomyelin (d18:0/16:0)*	9939965	**0.71**	0.0003	0.014
	behenoyl dihydrosphingomyelin (d18:0/22:0)*		**0.53**	0.0764	0.2342
	palmitoyl sphingomyelin (d18:1/16:0)	9939941	**0.69**	0.0003	0.014
	stearoyl sphingomyelin (d18:1/18:0)	6453725	**0.49**	8.22E-05	0.0066
	behenoyl sphingomyelin (d18:1/22:0)*		0.87	0.7677	0.5196
	tricosanoyl sphingomyelin (d18:1/23:0)*		**0.77**	0.0401	0.1728
	lignoceroyl sphingomyelin (d18:1/24:0)		0.79	0.1263	0.2787
	sphingomyelin (d18:1/14:0, d16:1/16:0)*	11433862	**0.7**	0.0725	0.2313
	sphingomyelin (d18:2/14:0, d18:1/14:1)*		0.55	0.1146	0.2667
	sphingomyelin (d18:1/15:0, d16:1/17:0)*		0.85	0.1666	0.3105
	sphingomyelin (d18:2/16:0, d18:1/16:1)*		0.81	0.1303	0.2787
	sphingomyelin (d18:1/17:0, d17:1/18:0, d19:1/16:0)		**0.66**	0.008	0.0867
	sphingomyelin (d18:1/18:1, d18:2/18:0)	6443882	**0.67**	0.003	0.0574
	sphingomyelin (d18:1/20:0, d16:1/22:0)*		0.62	0.1229	0.276
	sphingomyelin (d18:1/20:1, d18:2/20:0)*		0.71	0.1547	0.297
	sphingomyelin (d18:1/21:0, d17:1/22:0, d16:1/23:0)*		1.08	0.6293	0.4902
	sphingomyelin (d18:1/22:1, d18:2/22:0, d16:1/24:1)*		0.84	0.5881	0.4794
	sphingomyelin (d18:2/23:0, d18:1/23:1, d17:1/24:1)*		**0.71**	0.0782	0.2353
	sphingomyelin (d18:1/24:1, d18:2/24:0)*		**0.57**	5.56E-06	0.0012
	sphingomyelin (d18:2/24:1, d18:1/24:2)*		**0.61**	7.50E-06	0.0012
	sphingosine	5353955	0.75	0.4074	0.4033
	sphingosine 1-phosphate	5283560	1.01	0.7478	0.5156
	N-palmitoyl-sphingosine (d18:1/16:0)	5283564	**0.76**	0.087	0.2455
	N-stearoyl-sphingosine (d18:1/18:0)*	5283565	**0.53**	0.0079	0.0867
	glycosyl-N-palmitoyl-sphingosine		**0.56**	0.0004	0.0143
	glycosyl-N-stearoyl-sphingosine		**0.29**	0.0002	0.014
	lactosyl-N-palmitoyl-sphingosine		0.69	0.2724	0.3573
